# DELLA proteins positively regulate seed size in *Arabidopsis*

**DOI:** 10.1242/dev.201853

**Published:** 2023-08-09

**Authors:** Maria Dolores Gomez, Isabel Cored, Daniela Barro-Trastoy, Joaquin Sanchez-Matilla, Pablo Tornero, Miguel A. Perez-Amador

**Affiliations:** Department of Development and Hormonal Action in Plants, Instituto de Biología Molecular y Celular de Plantas (IBMCP), Universidad Politécnica de Valencia (UPV)-Consejo Superior de Investigaciones Científicas (CSIC), CPI 8E, Ingeniero Fausto Elio s/n, 46022 Valencia, Spain

**Keywords:** Ovule, Seed, Gibberellin, DELLA, *Arabidopsis*

## Abstract

Human and animal nutrition is mainly based on seeds. Seed size is a key factor affecting seed yield and has thus been one of the primary objectives of plant breeders since the domestication of crop plants. Seed size is coordinately regulated by signals of maternal and zygotic tissues that control the growth of the seed coat, endosperm and embryo. Here, we provide previously unreported evidence for the role of DELLA proteins, key repressors of gibberellin responses, in the maternal control of seed size. The gain-of-function *della* mutant *gai-1* produces larger seeds as a result of an increase in the cell number in ovule integuments. This leads to an increase in ovule size and, in turn, to an increase in seed size. Moreover, DELLA activity promotes increased seed size by inducing the transcriptional activation of *AINTEGUMENTA*, a genetic factor that controls cell proliferation and organ growth, in the ovule integuments of *gai-1.* Overall, our results indicate that DELLA proteins are involved in the control of seed size and suggest that modulation of the DELLA-dependent pathway could be used to improve crop yield.

## Teaser

**Highlighted Article:** In *Arabidopsis*, DELLA protein activity induces cell proliferation in ovule integuments that results in the development of larger ovules and, consequently, larger seeds.

## INTRODUCTION

Crop seeds are the main products for human and animal consumption, while also representing a significant source of biofuels (Savadi, 2018). For this reason, increasing the yield of seed crops is necessary to ensure both food security and biofuel production. Although seed yield is indeed a quantitative trait with genetic and environmental influences, it mostly depends on seed number and size. Therefore, since the origin of agriculture, plants have been selected for larger seed sizes throughout the course of domestication (Shomura et al., 2008; Fan et al., 2009). Therefore, uncovering the mechanisms regulating seed size is important to enhance the seed yield of crop plants. Over the past few years, investigations in *Arabidopsis thaliana* (*Arabidopsis* hereafter) and crops such as rice, maize and soybean have contributed to the current knowledge on seed development (Savadi, 2018; Li et al., 2019). However, the intricate network of molecular mechanisms and genetic factors controlling seed size are still unfolding.

In angiosperms, seed development begins with the double fertilization of the mature ovule, which triggers endosperm and embryo development (Chaudhury et al., 2001). One of the two sperm cells fuses with the egg cell to form the diploid embryo, while the other fertilizes the diploid central cell to generate the triploid endosperm. After fertilization, the maternal integuments of the ovule, surrounding the developing embryo and endosperm, undergo cell division and differentiation, thereby forming the seed coat (Haughn and Chaudhury, 2005). In *Arabidopsis*, ovule integuments consist of outer and inner integuments. The outer integument is composed of two cell layers (*oi1* and *oi2*), while the inner integument consists of three cell layers (*ii1*, *ii1′* and *ii2*) (Orozco-Arroyo et al., 2015). Thus, seed development is determined by the coordinated growth of the embryo, the endosperm and the seed coat. In summary, some factors act zygotically or maternally, or in both ways, to regulate seed growth, which extends from fertilization to 6 days after pollination (DAP) in *Arabidopsis* (Orozco-Arroyo et al., 2015).

Recent studies have revealed that, together with transcription factors (TFs), the ubiquitin pathway (Li and Li, 2014), G-protein signaling (Mao et al., 2010; Li et al., 2012), phytohormones and growth factors are engaged in the maternal control of seed size in *Arabidopsis* and rice (Li et al., 2019). The TFs identified in *Arabidopsis* include *TRANSPARENT TESTA GLABRA 2* (*TTG2*; Johnson et al., 2002), belonging to the WRKY family, which induces cell expansion in the ovule integument to promote seed growth (Johnson et al., 2002). In contrast, *APETALA 2* (*AP2*), a member of the AP2/EREBP (ethylene responsive element binding protein) family, controls seed size by limiting cell elongation in integuments (Ohto et al., 2005). Another key gene is *AINTEGUMENTA* (*ANT*), which encodes an AP2-domain TF with important roles in organ growth control, including the ovule (Klucher et al., 1996). Plants overexpressing *ANT* give rise to oversized organs, including flowers, ovules and seeds (Mizukami and Fischer, 2000; Barro-Trastoy et al., 2020). During ovule development, *ANT* acts maternally to control ovule and seed size (Li and Li, 2015).

Concerning hormones, those implicated in the maternal control of seed size include auxins, brassinosteroids (BRs) and cytokinins (CKs). Auxins regulate most plant growth and developmental processes, with the TFs AUXIN RESPONSE FACTORS (ARFs) acting as key regulators of auxin-mediated gene expression (Chandler, 2016). Among the 23 ARF genes in *Arabidopsis*, *ARF2* is known to control seed size by restricting cell proliferation of the ovule integument (Schruff et al., 2006). Thus, auxin signaling appears to set a physical restriction for seed growth and seed size via the ovule integuments. On the other hand, BRs positively regulate seed size by transcriptionally modulating specific seed developmental processes (Jiang et al., 2013). Thus, seeds of the BR-deficient mutant de-etiolated2 (*det2*) are smaller than those of wild-type plants due to a reduced endosperm volume and integument cell length. Through the activation of the TF BRASSINAZOLE-RESISTANT1 (BZR1), BRs upregulate the expression of *SHORT HYPOCOTYL UNDER BLUE1* (*SHB1*), *MINISEED 3* and *HAIKU2*, which are known positive zygotic regulators of seed size; however, BRs repress *AP2* and *ARF2*, which are negative maternal regulators of seed size (Jiang et al., 2013). Finally, CK-deficient or CK-insensitive mutants display larger seeds than wild-type plants (e.g. in the triple mutant of the three CK sensor histidine kinases AHK2, AHK3 and AHK4) (Riefler et al., 2006). Genetic analysis has shown that the CK-related increase in seed size is caused by both the maternal and/or endospermal genotypes (Riefler et al., 2006).

Gibberellins (GAs) are plant hormones involved in a large number of growth and development processes throughout the life cycle of plants (Sun, 2011; Gupta and Chakrabarty, 2013; Gallego-Giraldo et al., 2014; Gomez et al., 2016), including seed growth. Analyses of GA-deficient mutants in tomato, pea and *Arabidopsis* suggest that GAs are required for seed development (Groot and Karssen, 1987; Swain et al., 1997). In addition, the ectopic expression of a pea GA2oxidase2 (a GA-inactivating enzyme) in *Arabidopsis* results in seed abortion (Singh et al., 2002), whereas the overexpression of some GA-stimulated *Arabidopsis* (GASA) genes results in increased seed weight (Roxrud et al., 2007; Trapalis et al., 2017).

GAs regulate growth and development via the degradation of DELLA proteins, which act as GA signaling repressors. DELLA proteins belong to a subfamily of the plant-specific GRAS family of TFs (Sun, 2010). The bioactive GAs bind to the GID1 receptors, which promotes the binding of the DELLA protein to GID1. This GA-GID1-DELLA complex enables binding with F-box proteins, DELLA protein polyubiquitylation and its subsequent degradation by the 26S proteasome (Sun, 2011). The *Arabidopsis* genome encodes five DELLA proteins (GA-INSENSITIVE, GAI; REPRESSOR OF ga1-3, RGA; and RGA-LIKE (RGL) 1, RGL2 and RGL3). DELLA mutant proteins lacking the N-terminal DELLA regulatory domain (17 amino acid domain), as in the *gai-1* allele or the *pRGA:GFP-rgaΔ17* line of *Arabidopsis* (Peng et al., 1997; Dill et al., 2001), cannot be degraded by the GA-GID1 complex, resulting in constitutive DELLA activity and blockage of the GA-mediated gene expression response. Both high GA levels and the loss of function of DELLA proteins release GA responses, whereas low GA levels or GA-insensitive *della* mutants restrain GA responses (Sun, 2011; Vera-Sirera et al., 2015; Davière and Achard, 2016). DELLA proteins lack a canonical DNA-binding domain and thus mediate the transcriptional regulation of target genes through direct physical interaction with TFs and other regulatory proteins (Vera-Sirera et al., 2015; Davière and Achard, 2016).

Previous research from our group has suggested that gain-of-function *della* mutants (*gai-1* and *rgl2*Δ*17*), which promote GA response blockage, alter seed morphology (Gomez et al., 2016, 2019). In this work, we have characterized the seed phenotype of GA-related mutants and wild-type plants after pharmacological treatment with paclobutrazol (PBZ) to prevent GA synthesis. Our data indicate that DELLA activity promotes the production of larger seeds by increasing the cell number in both the outer and inner integuments of ovules, suggesting that DELLA proteins might maternally control seed size by inducing the increase of cell division in the integuments. The molecular mechanism of the DELLA-mediated control of ovule size could rely on *ANT* expression. ANT would mediate the function of DELLA in ovule development, with *ANT* as a direct target of GAI. Moreover, *ANT* overexpression mimics the ovule phenotype promoted by DELLA activity. Tackling the molecular mechanism by which DELLA proteins participate in the regulation of seed size would be useful for designing strategies to increase seed yield in crop plants.

## RESULTS

### GA-deficient and GA-insensitive *Arabidopsis* mutants produce large seeds

**Fig. 1.**
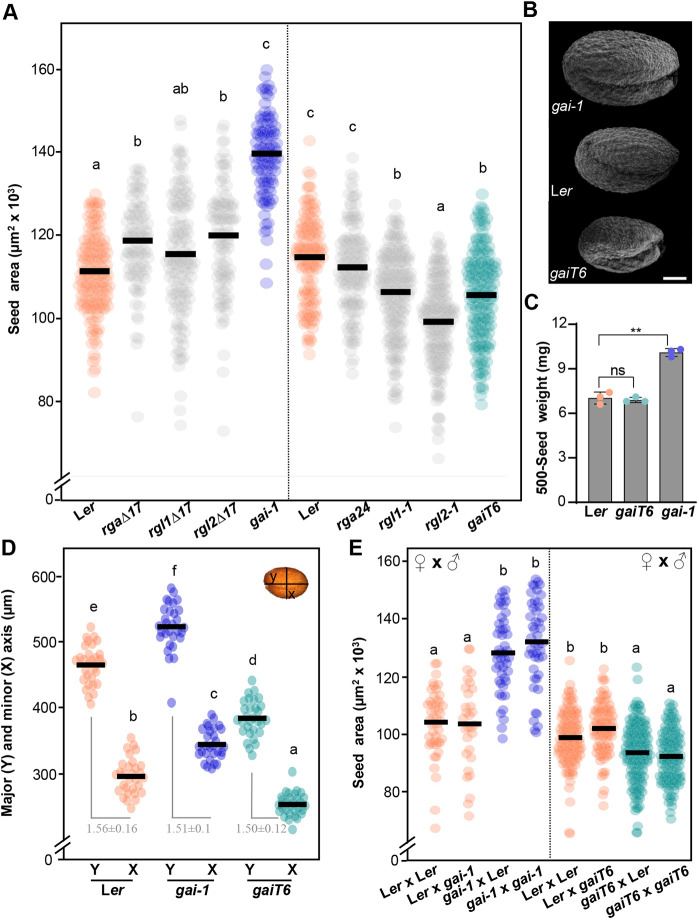
**DELLA proteins act maternally to positively regulate seed size.** (A) Seed area of L*er* (wild type), gain-of-function *della* mutants *rga*Δ*17*, *rgl1*Δ*17*, *rgl2*Δ*17* and *gai-1*, and loss-of-function *della* mutants *rga24*, *rgl1-1*, *rgl2-1* and *gaiT6*. (B) Scanning electron microscopy images of mature seeds of *gai-1*, L*er* and *gaiT6* plants. (C) Seed weight (mean±s.d. of 500 seeds) of L*er*, *gaiT6* and *gai-1* plants. Significant differences (Student's *t*-test) are indicated (**P<0.01); ns, not statistically significant. (D) Seed length (Y) and width (X) of L*er*, *gai-1* and *gaiT6* plants. The ratio of length to width (±s.d.) is shown in gray. (E) Seed area resulting from the indicated reciprocal crosses of L*er* with *gai-1* and *gaiT6*. The first genotype listed is the maternal parent (ovule) and the second is the paternal parent (pollen). (A,D,E) Dots represent data from individual seeds and horizontal lines represent the mean values (*n*>100 in A, *n*=30 in D and *n*≥30 in E). Different lowercase letters indicate a statistically significance difference, as determined by an ANOVA and a Bonferroni post-hoc test for multiple comparisons (*P*<0.05). Data that are not significantly different are marked with the same letter. Scale bar: 100 µm.

To determine the role of DELLA proteins in seed growth, we first examined the seed size of the gain-of-function *della* mutants *rga*Δ*17*, *rgl1*Δ*17*, *rgl2*Δ*17* and *gai-1* together with the loss-of-function mutants *rga24*, *rgl1-1*, *rgl2-1* and *gaiT6* (Fig. 1A,B). The dry mature seeds of gain-of-function *della* mutants were significantly larger than those of the wild type, except for *rgl1Δ17* seeds, which were only slightly larger than wild-type seeds. *gai-1* was associated with the greatest increase in size (∼25%), which correlated with an increase in seed weight of ∼42% (Fig. 1A-C). Interestingly, the seed number per *gai-1* fruit did not decrease when compared with the wild type, ruling out a possible compensatory mechanism between seed size and number in each fruit (Fig. S1A). The increased seed size is striking considering that *gai-1* plants exhibit a severe dwarf phenotype (Fig. S1A,B) (Koorneef et al., 1985; Peng et al., 1997). In contrast, null *della* mutants, with the exception of *rga24*, presented a significant decrease in seed size (Fig. 1A,B; Fig. S2A). Despite increasing or decreasing the seed size, DELLA activity did not modify the shape of seeds as the ratios between seed length and width in *gai-1* (gain-of-function mutant) and *gaiT6* (null mutant) were not different from the wild type (Fig. 1B,D).

Thereafter, we examined the seeds of mutants of the GA receptors *gid1a*, *gid1b* and *gid1c*, which show a lower sensitivity to GAs, and reduced degradation of DELLA proteins. Mutant *gid1a* and *gid1b* seeds were larger than the wild-type and *gid1c* seeds (Fig. S2B,D). This is consistent with the fact that both GID1a and GID1b receptors, but not GID1c, are expressed in ovules (Gallego-Giraldo et al., 2014). Finally, we verified that plants treated with paclobutrazol (PBZ), a GA biosynthesis inhibitor that reduces GA levels, thereby promoting DELLA protein accumulation, mimicked the seed phenotype of gain-of-function *della* mutants (Fig. S2C,E). In fact, the seeds of plants treated with PBZ were larger than those of any of the gain-of-function mutants, including *gai-1*. The greater increase in seed size after PBZ treatment could be due to the simultaneous stabilization of all DELLA proteins. In summary, our observations provide evidence of a positive correlation between DELLA activity and seed size in *Arabidopsis*. As *gai-1* showed the greatest seed size increase, we focused on the role of GAI by using *gai-1* and its corresponding null mutant *gaiT6* as genetic tools.

### DELLA proteins act maternally to control seed size

To determine whether GAI activity controls the seed size as a maternal o zygotic factor, reciprocal crosses between wild-type plants, *gai-1* mutants and *gaiT6* mutants were performed (Fig. 1E). Mature seeds produced by wild-type plants pollinated by *gai-1* or *gaiT6* pollen reached the same size as the seeds from wild-type plants hand-pollinated with wild-type pollen (i.e. both mutant pollens were unable to modify the seed size) (Fig. 1E). Consistent with these observations, no differences in size were detected in the seeds of *gai-1* and *gaiT6* plants pollinated with wild-type pollen when compared with those hand-pollinated with *gai-1* and *gaiT6* pollen (i.e. wild-type pollen did not alter the size of seeds in *gai-1* or *gaiT6* plants) (Fig. 1E)*.* In summary, the effect of the DELLA protein on seed size was observed only when the *gai-1* or *gaiT6* mutants acted as the maternal plant, regardless of the pollen genotype. This indicates that DELLA proteins control seed size by regulating the growth of the seed maternal tissue (i.e. the ovule integuments or/and the seed coat).

### DELLA activity promotes the cell proliferation of ovule integuments

**Fig. 2.**
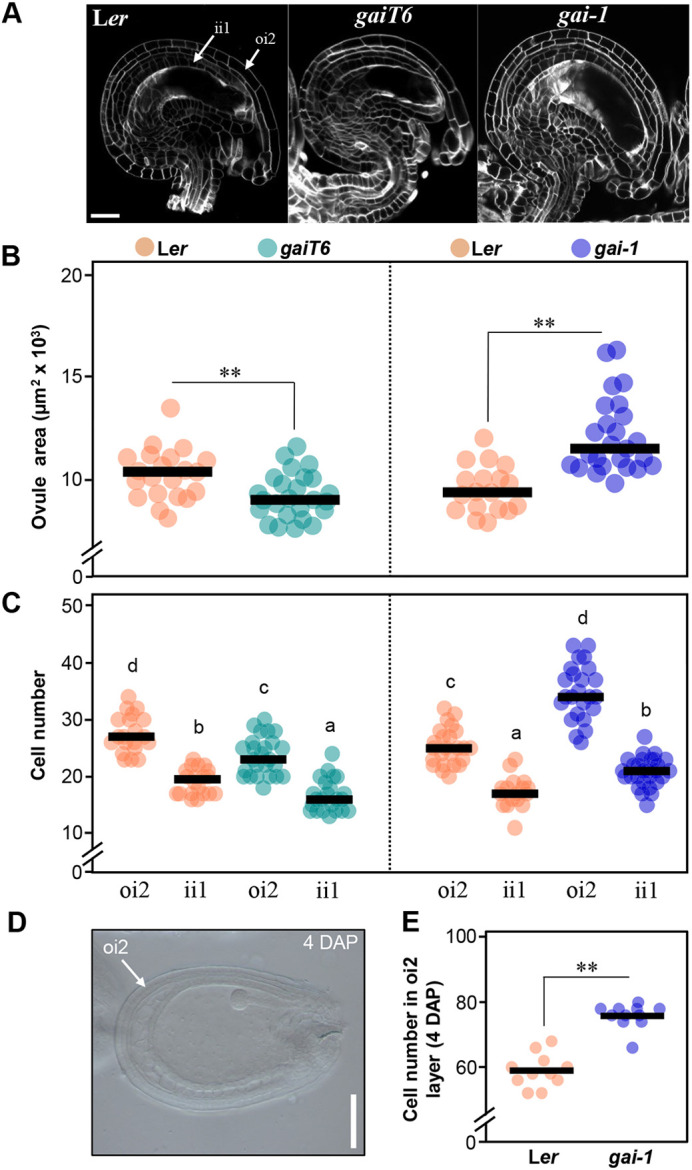
**GAI promotes ovule growth by increasing cell proliferation in integuments.** (A) Confocal images of representative mature ovules of L*er*, *gaiT6* and *gai-1* plants*.* (B) Ovule area of L*er, gaiT6* and *gai-1* plants (*n*≥20)*.* (C) Cell number in the outer layer of the outer integument (oi2) and the inner layer of the inner integument (ii1) of L*er*, *gaiT6* and *gai-1* mature ovules (*n*≥20)*.* (D) Image of a L*er* seed at 4 DAP observed by differential interference contrast (DIC) microscopy. (E) Cell number in the oi2 layer of L*er* and *gai-1* developing seeds at 4 DAP. (B,C,E) Dots represent data from individual ovules or cells and horizontal lines represent mean values. (B,E) Significant differences (Student's *t*-test) from the corresponding wild type are indicated (**P<0.01). (C) Different lowercase letters indicate a statistically significance difference, as determined by a one-way ANOVA and a Bonferroni post-hoc test for multiple comparisons (*P*<0.05). Data that are not significantly different are marked with the same letter. Scale bars: 20 µm in A; 100 µm in D.

After fertilization, the seed coat is formed from the differentiation of the integuments surrounding the ovule. Therefore, we asked whether DELLA protein activity affects early integument development and, consequently, the sizes of ovules and seeds. Indeed, the ovule size in mutant plants was significantly larger (*gai-1*) or smaller (*gaiT6*) than those from wild-type plants (Fig. 2A,B). Similarly, the ovule size of PBZ-treated plants was higher than those in mock-treated plants (Fig. S3A,B). Overall, ovule size phenotypes were consistent with the seed size of mutants or PBZ-treated plants. To determine how cell proliferation and cell expansion in the integuments in *gaiT6* and *gai-1* might contribute to ovule size, we analyzed the cell number in the outer oi2 and inner ii1 integument cell layers in both mutants (Fig. 2C). We found that *gaiT6* ovules had fewer cells in the oi2 and ii1 layers, which may explain their smaller size. In contrast, *gai-1* showed higher cell number in both the oi2 and ii1 layers when compared with the wild type. Furthermore, PBZ-treated plants also had more cells in the oi2 layer than mock-treated plants (Fig. S3C), thus mimicking the *gai-1* ovules. Next, we examined whether the increase in cell number in *gai-1* ovules was maintained during *gai-1* seed development (Fig. 2D,E). For this purpose, we analyzed the cell number of the seed coat epidermis (derived from the oi2 layer) of seeds at 4 DAP, considering that cell division is completed in the seed coat at this stage (Garcia et al., 2005). The cell number in the *gai-1* seed coat was also higher when compared with the wild type, which confirmed that the increase in seed size was due to the increase in cell proliferation during integument development. However, we cannot rule out the possibility that GAI also controls cell proliferation during seed coat differentiation. Finally, we also determined cell size and observed that, although *gai-1* has more cells in the integuments, these were smaller than those of the wild type (Fig. S4A-C)*.*

### DELLA proteins are expressed in developing ovules

**Fig. 3.**
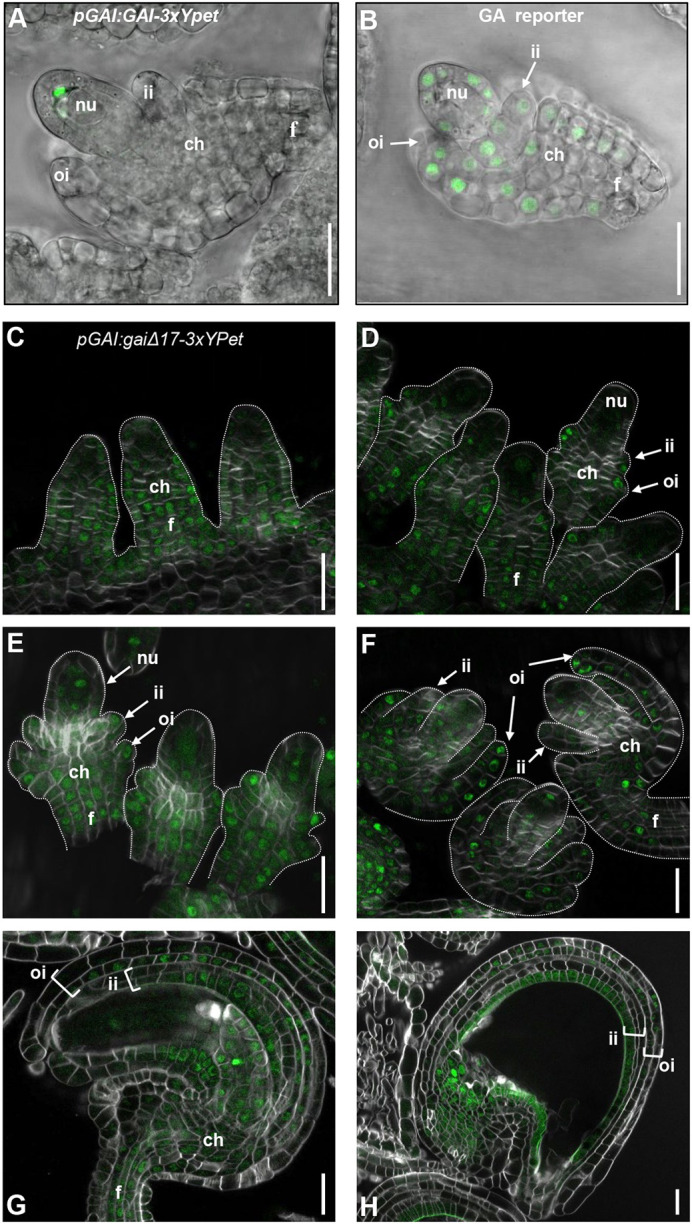
**GAI is expressed in developing ovules and seeds.** (A) Confocal images of GAI-3xYPet expression in ovules at stage 2-V. (B) GA-regulated reporter (GA HACR; Khakhar et al., 2018) expression in ovules at stage 2-V. Images correspond to an overlay of bright-field and fluorescence micrographs from fresh tissue. (C-H) Confocal images of the expression of gaiΔ17-3xYPet in the funiculus, chalaza and integuments of developing ovules (C, stage 2-I; D, 2-III; E, 2-IV; F, 2-V), mature ovules (G) and in the seed coat of developing seeds at 3 DAP (H). Ovules were cleared and cell walls were stained using Calcofluor White. The panels show the composite images of Calcofluor White and the *z*-stack projection of YPet fluorescence images. The dotted lines define the shape of the developing ovules. Confocal images are representative of several high-quality images obtained from three biological replicas. ch, chalaza; f, funiculus; ii, inner integument; oi, outer integument; nu, nucellus. Scale bars: 20 µm.

According to our data, a plausible scenario is that DELLA proteins in gain-of-function mutants or PBZ-treated plants might accumulate in developing integuments to enhance cell division. In previous work, we have reported that *GAI*, *RGA*, *RGL1* and *RGL2* genes are expressed in developing ovules (Gomez et al., 2016), and that stable YPet-rgl1Δ17 and YPet-rgl2Δ17 proteins driven by the respective endogenous promoters are also accumulated in ovule integuments (Gomez et al., 2019, 2020). These data are relevant because DELLA proteins undergo GA-dependent degradation; therefore, gene expression and protein presence do not always overlap. Furthermore, we have now verified that GFP-rgaΔ17 accumulates in developing ovules (Fig. S5A) using the *pRGA:GFP-rga*Δ*17* reporter line (Dill et al., 2001). We have also characterized the GAI protein localization during ovule development in detail (Fig. 3). For this, we generated transgenic lines expressing a wild-type version of the protein fused to 3xYPet (*pGAI*:*GAI-3xYPet*) flanked by the 15 and 5 kb genomic regions at the 5′ and 3′ ends of the locus, respectively. In wild-type ovules, GAI-3xYPet was detected within the nucellus, whereas no signal was observed in the chalaza, funiculus or integuments at any developmental stage (Fig. 3A). Using the GA HACR reporter line (Khakhar et al., 2018), we observed that active GAs were distributed throughout integuments, chalaza and funiculus, while integuments were developing (Fig. 3B). Therefore, the absence of GAI-3xYPet protein in these tissues during ovule development may be due to the accumulation of GAs, which triggers the degradation of DELLA proteins.

We also used the gain-of-function version *pGAI:gaiΔ17-3xYPet* described previously (Barro-Trastoy et al., 2022), which is identical to the wild-type version but with a 17 amino acid deletion of the DELLA domain mimicking that in the *gai-1* mutant. In this line, the gaiΔ17-3xYPet protein was clearly visible in ovules throughout development (Fig. 3C-H). At stage 2-I (Schneitz et al., 1995), before integuments are initiated, gaiΔ17-3xYPet was distributed in the funiculus and chalaza, from which the integuments will emerge (Fig. 3C). At stage 2-III, gaiΔ17-3xYPet was also expressed in emerging integuments (Fig. 3D). Localization remained in the same tissues at stages 2-IV and 2-V (Fig. 3E,F) until the ovule was mature (Fig. 3G). Upon fertilization, gaiΔ17-3xYPet was localized in the chalaza and the developing seed coat at 3 DAP (Fig. 3H). Overall, GAI activity could influence seed size through integument growth during ovule development and the initial stages of seed development.

### Expression of B-type cyclins is induced in integuments by DELLA activity

**Fig. 4.**
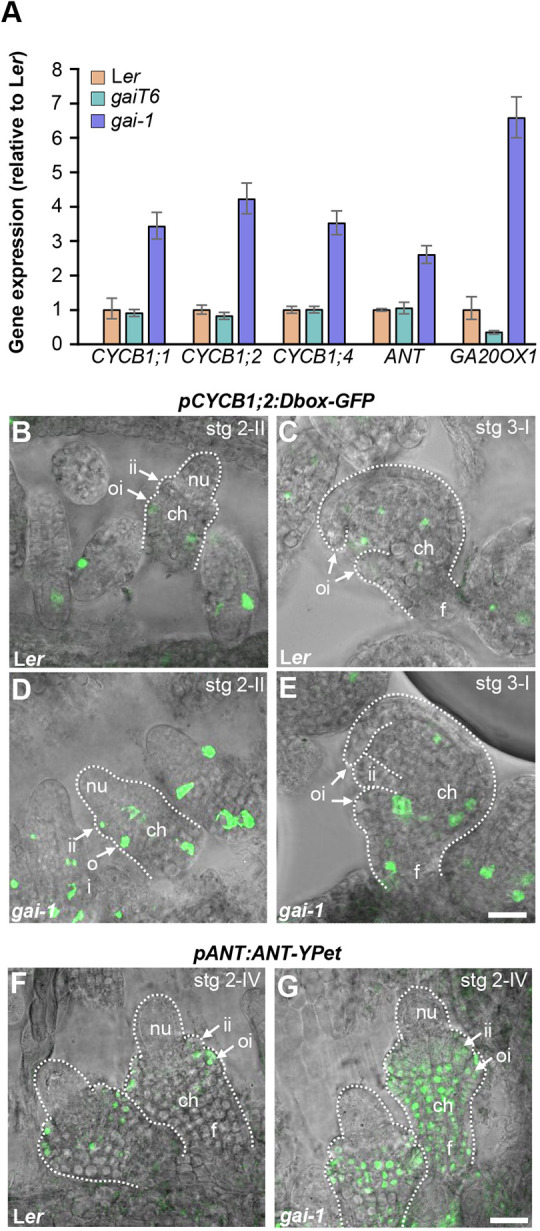
***ANT* and *CYC* gene expression is increased in *gai-1* ovules.** (A) Relative mRNA levels of *CYCB1;1*, *CYCB1;2*, *CYCB1;4*, *ANT* and *GA20OX1* (*At4g25420*) in siliques at 3 DAP of L*er, gaiT6* and *gai-1*. Data are normalized to *UBQ10* (*At4g05320*) in L*er*. Data are mean±s.d. of three replicates. The expression of *GA20OX1* was used to validate the result of the qPCR assay as it increases in *gai-1* and decreases in *gaiT6* (Rieu et al., 2008; Gallego-Bartolomé et al., 2011). (B-E) Confocal images of the localization of CYCB1;2-GFP in L*er* (B,C) and *gai-1* (D,E) ovules at stages 2-II (B,D) and 3-I (C,E). (F,G) Confocal images of the localization of ANT-YPet in L*er* (F) and *gai-1* (G) ovules at stage 2-IV. Panels show composite images of a bright-field image and a GFP (B-E) or YPet (F,G) *z*-stack projection. The dotted lines define the shape of the ovules. Confocal images are representative of several high-quality images obtained from three biological replicates. ch, chalaza; f, funiculus; nu, nucellus; ii, inner integument; oi, outer integument. Scale bars: 20 µm.

The increase and decrease of cell number in the integuments of *gai1* and *gaiT6* ovules, respectively, together with the *GAI* expression pattern, suggest that GAI can promote cell proliferation in the developing integuments of ovules. To test this, we examined the expression of B-type cyclins during ovule and seed development in the *gai-1* mutant. B-type cyclins are expressed shortly before and during mitosis, which makes them suitable markers for mitosis and cell proliferation (Colon-Carmona et al., 1999). Specifically, *CYCB1;1* (*At4g37490*), *CYCB1;2* (*At5g06150*) and *CYCB1;4* (*At2g26760*) were described to be expressed in ovules (Romeiro Motta et al., 2022). Interestingly, *CYCB1;1*, *CYCB1;2* and *CYCB1;4* expression level is increased in siliques at 3 DAP of *gai-1* (Fig. 4A). The number of GFP-positive nuclei in ovules of *gai-1* increased by 70% when compared with ovules from L*er* plants (5.09±1.77 and 3.03±1.31, respectively). Cyclin gene expression was also induced in *gai-1* inflorescences (Fig. S6A). Furthermore, using the reporter line p*CYCB1;2:Dbox-GFP* GFP (Merelo et al., 2022), we observed that *CYCB1;2* expression was higher in the developing ovules of *gai-1* when compared with the wild type (Fig. 4B-E). *gai-1* ovules showed the higher expression of CYCB1;2-GFP, with both an increased number of cells and higher signal intensity in the funiculus, chalaza and integuments (Fig. 4D,E). It should be noted that Fig. 4 presents ovules at developmental stages 2-II and 3-I, in which the integuments undergo a high growth rate (Vijayan et al., 2021). These analyses are consistent with an increase in cell proliferation in *gai-1* ovule integuments.

Expression assays using qPCR assays were performed on inflorescences containing developing ovules and on siliques with seeds at an early developmental stage (3 DAP) due to the impracticability of collecting only ovules. Therefore, we also specifically analyzed expression in ovules using confocal microscopy, mainly at the stages where most cell proliferation occurs in integuments.

### DELLA activity positively regulates *AINTEGUMENTA*

As described in the Introduction, several transcription and growth factors have been shown to control seed size by regulating cell proliferation in maternal tissues, including ANT (Klucher et al., 1996; Li and Li, 2015). Interestingly, *ANT* is also part of a list of bona fide gene targets of GAI obtained in a ChIP-Seq assay using *pGAI:gai*Δ*17-3xYPet* (Barro-Trastoy et al., 2022). ChIP-Seq analyses identified two putative indirect GAI-binding sites (through the interaction with a currently unidentified TF), located 3961 and 1256 bp upstream of the transcription start site of *ANT* (Fig. S7). Moreover, the ovule and seed phenotypes of *ANT*-overexpressing plants (Mizukami and Fischer, 2000) are similar to those of *gai-1*, with their flowers, ovules and seeds being larger than those of wild-type plants (Fig. S8). Thus, it is plausible that the molecular mechanism of the DELLA function relies in the direct transcriptional regulation of *ANT* during ovule development. To test this hypothesis, we checked the expression level of *ANT* in *gai-1* and *gaiT6* mutants. qPCR assays revealed that *ANT* expression level is increased in *gai-1* siliques at 3 DAP (Fig. 4A) and in inflorescences (Fig. S6A)*.* A second line of supporting evidence was provided by the *pGAI:gai-1-GR* transgenic line (Gallego-Bartolomé et al., 2011), which expresses a translational fusion between *gaiΔ17* and the rat glucocorticoid receptor under the control of the GAI promoter. Upon induction of *gai-1* by the dexamethasone (DEX) treatment of inflorescences, *ANT* expression was significantly increased (Fig. S6B). Furthermore, using the reporter line *pANT:ANT-YPet* (Barro-Trastoy et al., 2020), we observed that *ANT* expression was clearly higher in the chalaza, integuments and funiculus of developing *gai-1* ovules when compared with the wild type (Fig. 4F,G). Thus, the activation of cell proliferation during ovule development by GAI may involve the increased expression of *ANT* in this organ, with ANT being a putative direct target of GAI. Taken together, these results suggest that DELLA activity would increase seed size by activating the expression of *ANT*, which would increase integument cell proliferation and thus ovule size. Unfortunately, no genetic assays could be performed to further confirm these results as *ANT* null mutants do not produce viable ovules or seeds.

### DELLA proteins do not modify seed structure and composition

**Fig. 5.**
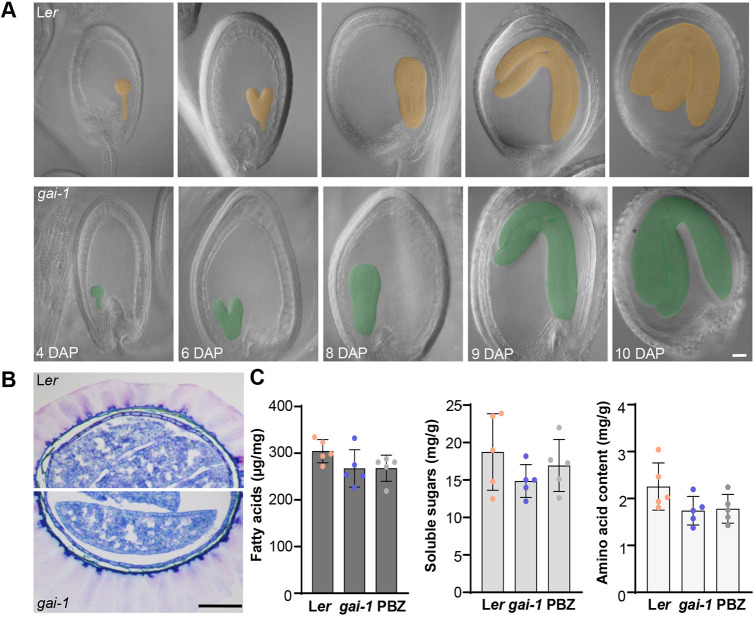
***gai-1* seeds show no changes in morphological development or metabolic composition.** (A) Embryo development in L*er* and *gai-1* plants. L*er* and *gai-1* cleared seeds at 4, 6, 8, 9 and 10 DAP were observed using DIC microscopy. The embryos were colored (orange for L*er* and green for *gai-1*) to facilitate visualization. (B) Semi-thin sections of L*er* and *gai-1* dry seeds embedded in resin showing the external mucilage. (C) Fatty acid, sugar and amino acid content in dry seeds of L*er*, *gai-1* and L*er* upon treatment with 1 µM PBZ. Dots represent individual values for each of the five biological replicates. One-way ANOVA and Tukey's multiple comparison tests were performed; no statistically significant differences were found. Scale bars: 50 μm in A; 100 μm in B.

An important question related to the possible use of DELLA proteins as a biotechnological tool to improve seed size in crops is whether embryo development, structure and metabolic composition are also impacted (i.e. whether or not DELLA proteins are involved in developmental or differentiation processes other than seed growth). Fig. 5A shows that wild-type and *gai-1* embryos developed similarly, with the same timing. In addition, no differences were detected in the structure of the seed coat, including the mucilage, between *gai-1* and wild-type dry mature seeds (Fig. 5B). That is, the increase in seed size by enhanced DELLA activity does not cause modifications to seed coat structure and morphology. Regarding metabolic composition, we compared the amount of fatty acids, soluble sugars (sucrose, fructose and glucose) and free amino acids in dry mature seeds from wild-type, *gai-1* and PBZ-treated plants (Fig. 5C). Similar levels of the three types of compounds were detected in all analyzed seeds. Taken together, these data suggest that modulating DELLA activity only impacts seed size, with no alterations on their development, anatomy or metabolic content.

## DISCUSSION

Seeds ensure the survival and dispersal of plants, and represent the basis of human and animal nutrition. In recent years, significant efforts have been made to understand how seed size is regulated by the signals of maternal and zygotic tissues (Li et al., 2019). Hormones and TFs are integrated into a complex genetic network that regulates seed size in a maternal manner. In this context, auxins, BRs and CKs have been shown to regulate seed size in *Arabidopsis* (Li and Li, 2015; Orozco-Arroyo et al., 2015; Li et al., 2019). Our work unveils that GAs do not only influence proper seed growth, but that they are also leading players in the control of seed size via DELLA function in maternal tissues.

The repression of GA signaling (e.g. in the gain-of-function *DELLA* mutants, in *gid1* receptor mutants or in PBZ-treated plants) results in larger seeds, whereas the partial activation of GA responses (e.g. in single loss-of-function *DELLA* mutants) leads to smaller seeds (Fig. 1). In the case of GAI (the DELLA protein that has the greatest effect), stabilization also increased seed weight without affecting its metabolic composition (fatty acid, sugar or amino acid content) or altering seed morphology or the number of seeds per silique (Figs 1 and 5). These characteristics make GAI a potentially powerful biotechnological tool for increasing seed crop yield.

It is well established that GAs promote stem, leaf and root cell proliferation and expansion by inducing the degradation of growth-repressing DELLA proteins (Achard et al., 2009; Davière et al., 2014; Serrano-Mislata et al., 2017). As a result, mutants that stabilize DELLA proteins, such as *gai-1*, are dwarf plants. Strikingly increased DELLA activity results in larger rather than smaller seeds, indicating that DELLA proteins have an opposite function in seed development compared with other plant organs. As DELLA activity positively regulates biotic and abiotic stress resistance while repressing cell division and expansion (Wild et al., 2012; Davière and Achard, 2016; De Vleesschauwer et al., 2016; Thomas et al., 2016; Shi et al., 2017), we speculate that the DELLA-dependent promotion of seed growth might be part of a similar trade-off mechanism that aims to ensure optimal reproductive development and improve the next generation. Further experiments would be required to confirm this hypothesis.

Through reciprocal crosses between wild-type plants and *gai-1* and *gaiT6* mutants, we determined that DELLA proteins induce the growth of the seed from maternal tissue (Fig. 1); i.e. they regulate seed size by increasing the integument growth of ovules and developing seeds (Fig. 2). The number of outer and inner integument cells in *gai-1* mutant ovules and seeds was significantly higher when compared with the wild type, indicating that GAI regulates seed size by promoting cell proliferation associated with an elevated CYCB1;2 activity in the maternal integuments (Figs 2 and 4). As cells in the integuments mainly undergo expansion after fertilization (Garcia et al., 2005), the number of cells in the *gai-1* ovule integuments is expected to determine the final size of the seed coat. Furthermore, although mutations that act maternally to produce larger seeds often display a prolonged period of seed coat growth and delayed embryo development in *Arabidopsis* (Ohto et al., 2009; Fang et al., 2012), *gai-1* embryo development was not delayed when compared with the wild type (Fig. 5).

Similar to GAI, other genetic factors induce cell proliferation in the integuments, resulting in larger seeds. These include the TFs BZR1 and ANT (Mizukami and Fischer, 2000; Jiang et al., 2013), and the cytochrome P450 proteins KLUH (KLU, CYP78A5) and EOD3 (CYP78A6) (Adamski et al., 2009; Fang et al., 2012). Some of these genetic factors could be GAI targets or interactors during integument development. BZR1 is a well-known DELLA interactor, and GAs and BRs co-regulate many aspects of plant development in both a cooperative and antagonistic manner (De Vleesschauwer et al., 2012; Unterholzner et al., 2015; Barro-Trastoy et al., 2020). Despite this, ovules of the gain-of-function *bzr1-1D* mutant have increased the proliferation and expansion of integument cells (Jiang et al., 2013), which differs from the *gai-*1 ovule phenotype in which cells decrease in size (Fig. S4). In addition, ovules of the gain-of-function *eod3-1D* mutant have a similar phenotype to *bzr1-1D* (Fang et al., 2012). It is possible that the control of cell expansion and cell division in the integument by DELLA and BZR1 proteins relies on two different molecular mechanisms (trans-activation versus sequestration). Only ANT and KLU proteins behave similarly to GAI during integument development. Plants overexpressing ANT or KLU show increased integument cell number at the expense of decreasing the cell size similar to the *gai-1* mutant (Mizukami and Fischer, 2000; Adamski et al., 2009). This observation, together with the fact that *ANT* was identified as a potential target of GAI through a ChIP-Seq analysis (Barro-Trastoy et al., 2022), strongly suggests that *ANT* may participate in the GAI pathway responsible for the GA-dependent regulation of seed size. In fact, *ANT* expression significantly increases in the inflorescences of *gai-1* mutant and upon induction with DEX in the *4xdella pGAI:gai-1-GR* transgenic line (Fig. 4; Fig. S6). Additionally, ANT-YPet protein level is greater in *gai-1* developing ovules. All these results reinforce the notion that GAI could trigger *ANT* induction during ovule development to maternally control *Arabidopsis* seed size. This scenario is further supported by the increase of cell divisions in *gai-1* integuments, as reflected by the observed *CYCB1;2* expression, given that the gain of ANT function allows cells to proliferate for a longer period than normal, thereby causing organ enlargement (Mizukami, 2001). Adding an extra layer of complexity, ChIP-Seq experiments revealed that ANT could bind to regions upstream of the *GAI* CDS and that GAI would be differentially expressed after ANT-GR activation (Krizek et al., 2020); therefore, both genes could co-regulate each other in a positive-feedback mechanism.

**Fig. 6.**
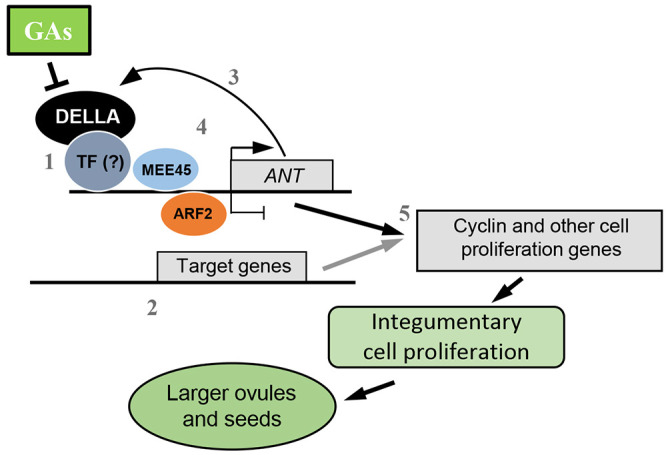
**Proposed working model of how DELLA activity regulates ovule and seed size in *Arabidopsis*.** GAs mediate DELLA protein degradation, which is a positive factor in the promotion of cell proliferation in integumentary cell layers during ovule development. DELLA proteins interact with an unknown TF to positively regulate the expression of *ANT* (1). DELLA may also interact with other TFs to regulate other target genes (2). ANT upregulates *GAI* directly by binding to its promoter (3). In addition, other TFs, such as MEE45 or ARF2, can also directly regulate the expression of *ANT* (4). Altogether, the coordinated expression of cyclin and other genes regulate cell proliferation in ovule integuments (5). As a result, the cell number in integuments is increased, which leads to larger ovules and seeds.

It is most likely that *ANT* expression is not solely regulated by GAI. Previous studies have shown that the B3 domain TFs ARF2 and MATERNAL EFFECT EMBRYO ARREST 45 (MEE45) have ANT as a common target (Meng et al., 2015; Li et al., 2021). These TFs exert opposite transcriptional regulatory functions and thus cause inverse effects on seed size. ARF2 binds to the *ANT* promoter and negatively controls seed growth by restricting integument cell proliferation (Schruff et al., 2006), whereas MEE45 directly induces *ANT* expression and thus promotes integument cell proliferation and increases seed size (Li et al., 2021). These findings, combined with the fact that GAI could also be an activator of *ANT*, highlight the multiple signaling pathways that converge at ANT for the maternal control of seed development. Based on our findings and the previously described data, we postulate a working model for the mechanism of DELLA function in ovule and seed size (Fig. 6). Notably, GAs negatively regulate DELLA protein stability. DELLA proteins would regulate the expression of *ANT* (and other putative targets) via interaction with an unknown TF(s) that confers target specificity; ANT activity would in turn increase cell proliferation. Additionally, through a feedback mechanism, ANT would directly upregulate *GAI* by binding to its promoter to ensure a tighter degree of control. Finally, *ANT* expression is also regulated by other TFs, such as MEE45 or ARF2.

In most agricultural crops, seeds are the main product for harvesting. Thus, an increase in seed size would be beneficial for improving seed crop yield. In this work, we describe a previously unreported role for DELLA proteins as positive regulators of ovule and seed size. Further efforts would be necessary to fully determine the genetic and molecular mechanisms through which DELLA proteins are involved in seed size control and to address whether elements of this DELLA-dependent pathway could be used to engineer larger seed size in crops.

## MATERIALS AND METHODS

### Plant materials and growth conditions

*Arabidopsis thaliana* plants Landsberg *erecta* (L*er*) or Columbia-0 (Col-0) were used as wild-type lines. All mutants and reporter lines have been previously described. In the Ler background, we have used *gai-1* (Koorneef et al., 1985), *gaiT6* (Peng et al., 1997), *rga24* (Silverstone et al., 1998), *rgl1-1* and *rgl2-1* (Lee et al., 2002) mutants, and the lines *pRGA:GFP-rga*Δ*17* (Dill et al., 2001), *4xdella pGAI:gai-1-GR* (Gallego-Giraldo et al., 2014), *pRGL2:YPet-rgl2*Δ*17* (Gomez et al., 2019), *pRGL1:YPet-rgl1*Δ*17* (Gomez et al., 2020), *pANT:ANT-YPet* (Barro-Trastoy et al., 2020), *pCYCB1;2:Dbox-GFP* (Merelo et al., 2022) and *pGAI:gai*Δ*17-3xYPet* (Barro-Trastoy et al., 2022). In the Col-0 background, we have used *gid1a*, *gid1b* and *gid1c* (Griffiths et al., 2006), *gai-1* (Barro-Trastoy et al., 2020), *35S:ANT* (Barro-Trastoy et al., 2020), and GA HACR (hormone-activated Cas9-based repressor) plants with the PHD6 genotype (Khakhar et al., 2018). We generated the *gai-1 ANT-YPet* plants by crossing *gai-1* with the *pANT:ANT-YPet* line. F3 homozygous plants were selected by PCR-based genotyping and/or antibiotic or herbicide resistance. All primers used for genotyping are listed in Table S1. Reagents and primers were purchased from Sigma-Aldrich or Integrated DNA Technologies, respectively, unless otherwise stated.

Seeds were surface-sterilized in ethanol, plated onto ½ Murashige and Skoog medium (Murashige and Skoog, 1962), incubated at 4°C for 3-4 days in darkness, and transferred to a growth chamber at 24°C in a long-day photoperiod (16/8 h) for 7-10 days. Seedlings were then transferred to soil (a 2:1:1 mix of peat moss, vermiculite and perlite) and grown in a chamber at 22°C in a long-day photoperiod. PBZ treatment was applied by watering the plants every other day with 1 µM PBZ (Duchefa Biochemie) starting at bolting. A stock solution of 10 mM PBZ was prepared in acetone. DEX was applied onto inflorescences of the line *4xdella pGAI:gai-1-GR* by spraying a 5 µM solution, and inflorescences were collected 24 h after treatment. DEX stock solution was made at 10 mM in ethanol.

To conduct reciprocal crosses, the flower buds of L*er*, *gai-1* and *gaiT6* were hand-emasculated 1 day before anthesis, and pistils were hand-pollinated the next day with mature pollen from one of the three different genotypes. Fruits (only one fruit per plant) were collected at maturity and seed size was measured (*n*≥30 fruits per pollination). All experiments were repeated three times, with similar results.

### Generation of the transgenic *pGAI:GAI-3xYPet* reporter line

The *pGAI:GAI-3xYPet* line was generated by recombineering (Brumos et al., 2020) in a manner similar to the gain-of-function line *pGAI:gaiΔ17-3xYPet* described by Barro-Trastoy et al. (2022). All primers used are listed in Table S2. The construct was used to transform L*er Arabidopsis* plants by *Agrobacterium*-mediated floral dipping (Clough and Bent, 1998), and transgenic plants were selected in ammonium glufosinate to obtain homozygous lines segregating as a single locus. To check whether GAI-3xYPet behaves like a DELLA protein, the effect of GAs and PBZ on stability of GAI-3xYPet fusion protein was tested in primary roots of seedlings. Transgenic plants were grown on MS plates for 2 days and treated with 100 µm GA_4+7_ or with 100 um PBZ for 24 h and 48 h, respectively (Fig. S5B). YPet fluorescence was observed using confocal microscopy as indicated below.

### Morphological and cellular analysis

To determine seed size, dry seeds were photographed on white paper under a MZ16F stereomicroscope (Leica) using a DMC6200 digital camera (Leica). The area, length and width of seeds were measured from the images using ImageJ software (https://imagej.nih.gov/ij/). Seed size was inferred from the obtained images as the medial seed area in a 2D projection. From each genotype, at least 100 seeds from 20 different siliques (one silique per plant between positions 10 and 20 of the main inflorescence) were used. Average seed weight was determined by weighing dry seeds in three batches of 500 seeds each using an AB54 analytical balance (Mettler Toledo).

To measure the ovule area, cell number and cell size, mature ovules from pistils at the anthesis stage were cleared and visualized with confocal microscopy, as indicated below. Fig. S9 presents an image illustrating how the ovule size, the cell number in the integuments and the average cell size (integument length/integument cell number) from the different plants were estimated using ImageJ software. Statistical tests were performed using GraphPad Prism 8.0.2 and Statgraphics 18.1.13 software, as well as online web statistical calculators (www.astatsa.com).

### Histological procedures

Seed development was studied using chloral hydrate clearing and differential interference contrast microscopy (DIC). Siliques at different developmental stages were fixed in ethanol:acetic acid (9:1, v/v) for 3 h, washed with 90% ethanol and then cleared with a chloral hydrate solution (mixture of chloral hydrate:water:glycerol 8:2:1, w/v/v) for at least 3 days. Images were recorded using an Eclipse E600 microscope (Nikon) equipped with DIC optics and a DS-Ri1 digital camera (Nikon).

For the histological analysis of seed coats, dry seeds were fixed overnight at 4°C in 4% (w/v) p-formaldehyde in 0.1 M sodium phosphate (pH 7.2) with 0.05% (v/v) of Tween 20 and dehydrated in ethanol series. Seeds were then infiltrated in Technovit 7100 resin (Heraeus Kulzer), sectioned in an Ultracut E microtome (Reichert Jung) at 3 µm, and stained in 0.02% Toluidine Blue O, as described by Gómez et al. (2004). Images were captured using a DM5000 microscope (Leica).

### Confocal laser scanning microscopy

Confocal microscopy was used to study the expression of reporter lines in developing ovules. To measure the ovule area and study the expression of gaiΔ17-3xYPet, samples were previously cleared. For this, inflorescences and mature ovules were fixed in vacuum for 1 h in 4% (w/v) p-formaldehyde in 0.1 M sodium phosphate (pH 7.2). After fixation, samples were cleared with ClearSee solution (Kurihara et al., 2015) for a least 1 week, and then stained with 0.01% (w/v) Calcofluor White (Ursache et al., 2018) before confocal microscope observation. GAI-3xYPet, ANT-YPet, CYCB1;2-GFP and GA reporter signals were observed in fresh tissues.

Images were captured using an LSM 780 confocal microscope (Zeiss). YPet fluorescent protein was observed with excitation at 514 nm and detection at 520-550 nm. Calcofluor White was observed with excitation at 405 nm and detection at 430-480 nm in grayscale. GA HACR ovules were analyzed via the detection of Venus fluorescent protein with excitation at 488 nm and detection at 510-550 nm. The identity of fluorescence signals was confirmed using a λ-scan. For each analysis, several high-quality images were obtained from three biological replicas.

### Scanning electron microscopy (SEM)

Dry seeds were mounted on SEM stubs, coated with gold for 90 s in a SCD 005 sputter coater (BAL-TEC), and photographed using a Field Emission Scanning Microscopy ULTRA 55 microscope (Zeiss Oxford Instruments) using incident electron energy of 2 kV. The images were acquired at the electron microscopy facility of Universitat Politecnica de Valencia (UPV).

### Quantitative RT-PCR (qPCR)

For qPCR analysis, dissected main inflorescences (removing open flowers) and siliques at 3 DAP were collected and flash frozen in liquid nitrogen. Total RNA was extracted using a NucleoSpin RNA Plant kit (Macherey-Nagel) according to the manufacturer's instructions. cDNA was synthesized from 1 µg of total RNA using a PrimeScript 1st Strand cDNA Synthesis kit (Takara Bio Inc). The resulting cDNA was diluted 1:9 in deionised H_2_O and 1 µl of the resulting dilution was used in the qPCR reaction. qPCR was performed in a 7500 Fast Real-Time PCR System (Applied Biosystems) with SYBR premix ExTaq (Tli RNAase Plus) Rox Plus (Takara Bio). Expression levels were normalized to *UBQ10* (*At1g05320*) (Czechowski et al., 2005) and analyzed by the comparative ΔΔCt method (Schmittgen and Livak, 2008) to the values in the wild-type or mock-treated plants. Three biological replicates with three technical replicates each were processed. The primers used for qPCR are listed in Table S1.

### Metabolite analysis

Soluble sugars and amino acids were analyzed as described by Minebois et al. (2020), using 10 mg of dry seeds per sample. For fatty acid extraction and methylation, 1 ml of 5% H_2_SO_4_ in methanol, 300 µl of toluene, 5 µl of 1% (w/v) butylhydroxytoluene in methanol and 5 µl of 1% (w/v) heptadecanoic acid in chloroform as an internal standard were added to 10 mg of seeds. Samples were incubated at 85°C for 90 min, then 1.5 ml of 0.9% (w/v) NaCl and 1 ml of hexane were added. After vortexing and brief centrifugation, the upper phase (organic) was extracted for injection in the gas chromatograph. The GC-MS conditions used are described in Minebois et al. (2020). Metabolite analyses were performed at the Instituto de Biologia Molecular y Celular de Plantas (UPV-CSIC, Valencia, Spain) Metabolomics Platform.

## Supplementary Material

10.1242/develop.201853_sup1 Supplementary information
