# Supplementary material for: DELLA proteins positively regulate seed size in Arabidopsis
Source: Development. 2023 Aug 9;150(15):dev201853. doi: 10.1242/dev.201853 (PMC10445750; doi:10.1242/dev.201853)
Supplement: Supplementary information [file develop-150-201853-s1.pdf]

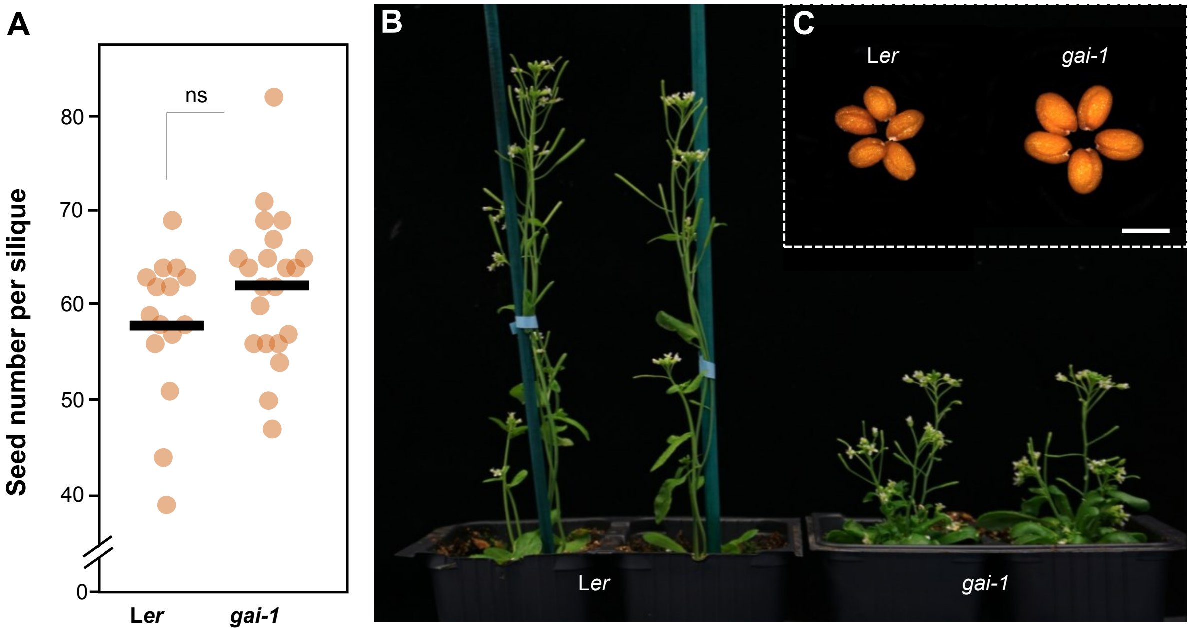

Fig. S1. *gai-1* plants exhibit a dwarf phenotype but larger seeds when compared to L*er* plants, with no change in the number of seeds per fruit. (A) Seed number per silique in L*er* and *gai-1* plants. (B and C) Images of flowering plants (B) and mature seeds (C) of L*er* and *gai-1*. Dots represent data from individual siliques, and the horizontal line represents the mean value (n≥15). The difference between pairs was determined by a Student's *t*-test. ns, not statistically significant. Scale bar represents 500 µm.

Development • Supplementary information

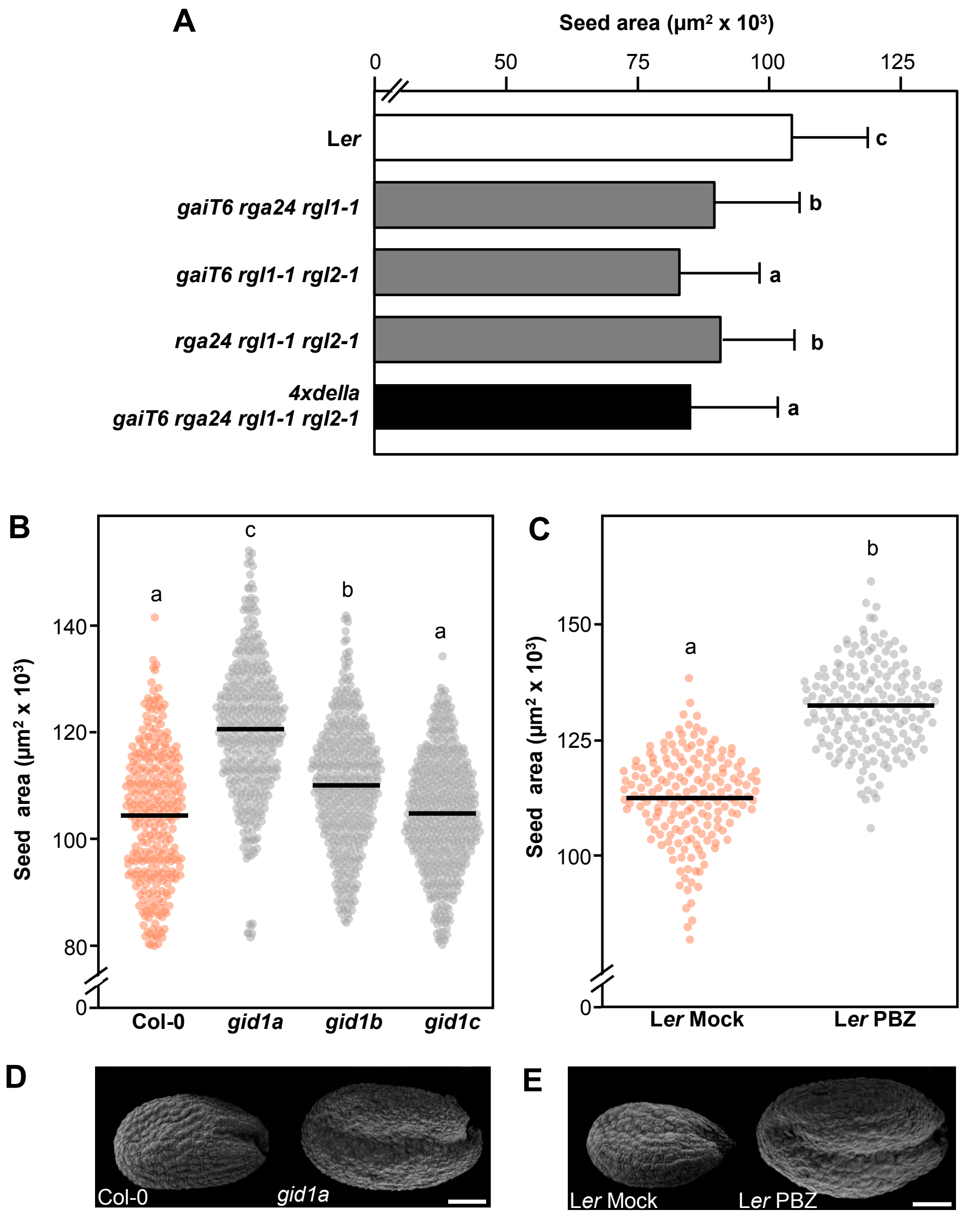

Fig. S2. *della* and *gid1* mutants and PBZ-treated plants have altered seed size. (A) Seed area of L*er* (WT) and triple *della* mutants *gaiT6 rga24 rgl1-1*, *gaiT6 rgl1-1 rgl2-1*, and *rga24 rgl1-1 rgl2-1*, and the *4xdella* (*gaiT6 rga24 rgl1-1, rgl2-1*). (B) Seed areas of Col-0 (WT) and *gid1a*, *gid1b,* and *gid1c* mutants. (C) Seed area of L*er* upon treatment with mock or 1 µM PBZ. (D) Scanning electron microscopy (SEM) images of mature seeds of Col-0 and the *gid1a* mutant. (E) SEM images of L*er* seeds upon treatment with mock or 1 µM PBZ. In (A), data are mean values ± SD (n≥100). In (B) and (C), dots represent data from individual seeds (n>100) and horizontal lines represent mean values. Lowercase letters indicate statistical significance, as determined by a one-way ANOVA and a Bonferroni post hoc test for multiple comparisons ($P<0.05$). Data that are not significantly different are marked with the same letter. Scale bar represents 100 µm.

Development • Supplementary information

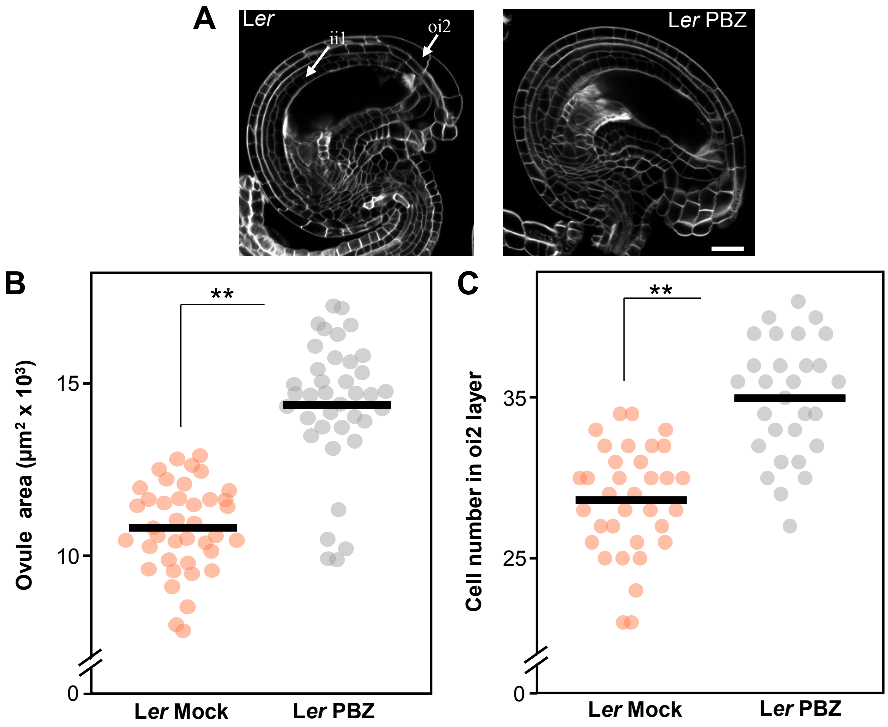

Fig. S3. GA biosynthesis inhibition triggers an increase in ovule size. (A) Confocal images of representative mature ovules of L*er* upon treatment with mock or 1 µM PBZ. (B and C) Mature ovule area (B) and cell number in the oi2 layer (C) of mature ovules of L*er* upon treatment with mock or 1 µM PBZ (n≥30). Dots represent data from individual ovules (left panel) or cells (right panel), and horizontal lines represent mean values. Significant differences (Student's *t*-test) with the corresponding mock are indicated (** *P*<0.01).

Development • Supplementary information

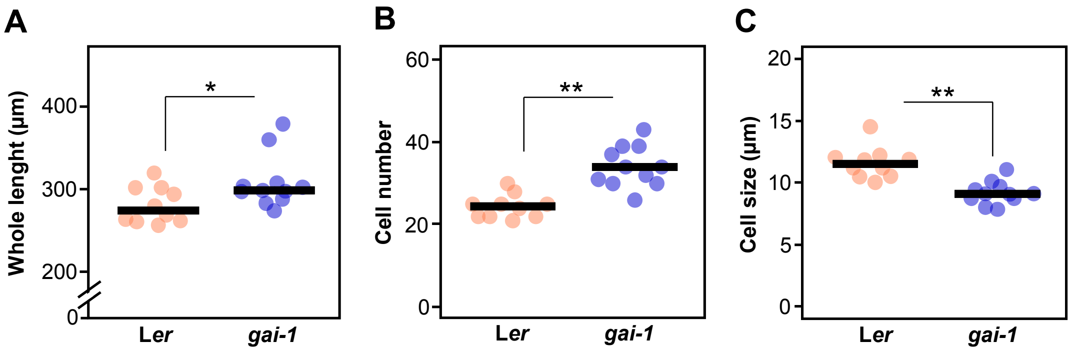

Fig. S4. *gai-1* integument cells are more numerous but smaller than those of L*er*. (A–C) Whole length (A), cell number (B), and cell size (C) of the oi2 integument layer of mature ovules of L*er* and *gai-1*. Dots represent data from individual ovules or cells and the horizontal lines represent mean values. Significant differences (Student's *t*-test) with the correspondent WT are indicated (* P<0.05, ** P<0.01).

Development • Supplementary information

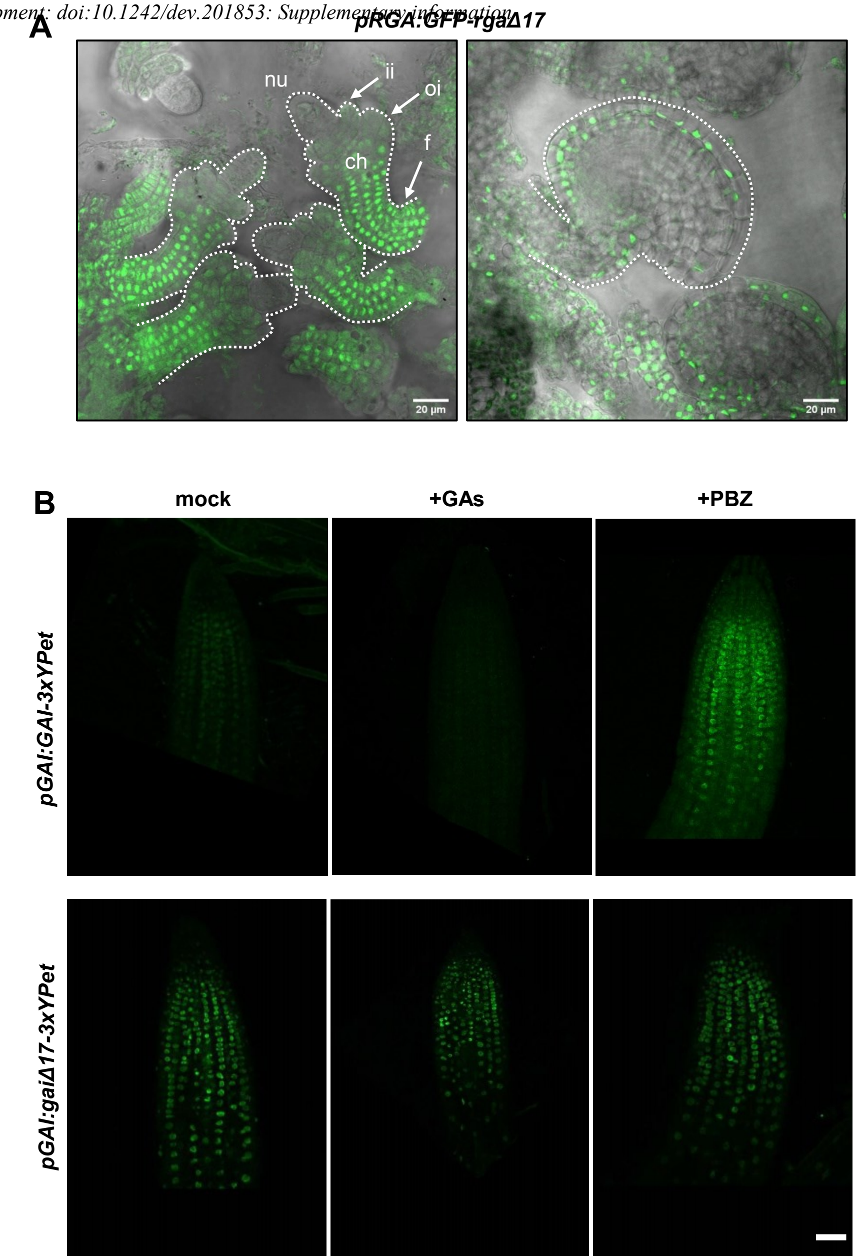

Fig. S5. (A) GFP-rgaΔ17 is present in developing ovules and (B) effects of GA and PBZ treatment on the stability of GAI-3xYPet and gaiΔ17-3xYPet proteins. (A) GFP-rgaΔ17 localization from the *pRGA:GFP-rgaΔ17* line in the funiculus, chalaza, and integuments of ovules at stages 2-IV (left panel) and 3-IV (right panel). Panels show the composite image of Calcofluor White and GFP fluorescence. The dotted lines define the shape of the ovules. ch, chalaza; f, funiculus; ii, inner integument; oi, outer integument; nu, nucellus. (B) GAI-3xYPet is localized in the root nuclei (mock), but it is degraded in the presence of 100 µM GA_{4+7} (+GA) or stabilized in the presence of 100 µM PBZ (+PBZ). In contrast, gaiΔ17-3xYPet levels are not altered upon GA or PBZ treatments. Confocal images are representative of several high-quality images obtained from three biological replicas. Scale bars represent 20 µm in A and B.

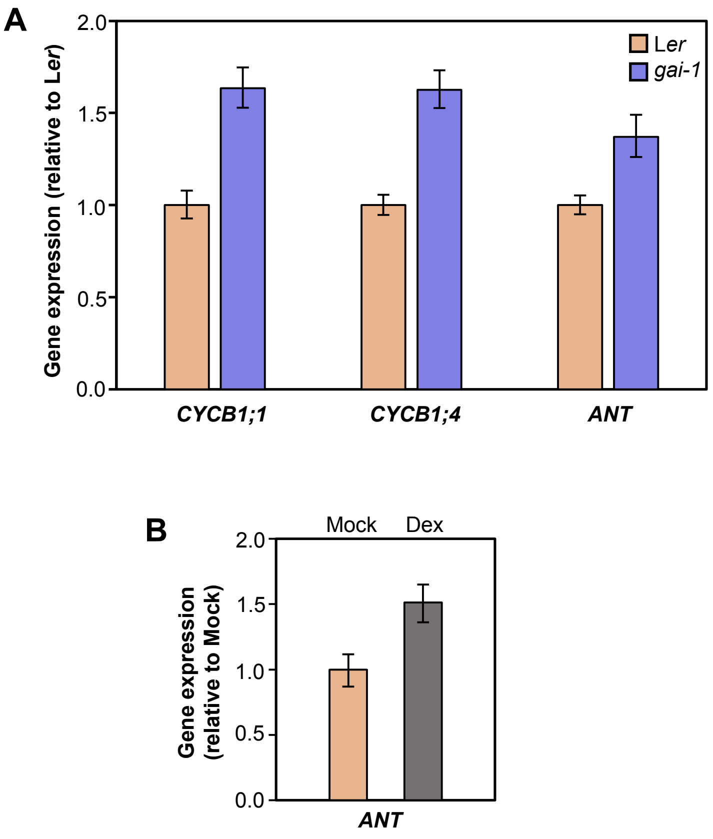

Fig. S6. *CYCB1;1, CYCB1;4,* and *ANT* genes are upregulated when GAI protein is stabilized. (A) Relative mRNA levels of *CYCB1;1, CYCB1;2, CYCB1;4*, and *ANT* in the inflorescences of L*er* and *gai-1*. (B) Relative mRNA levels of *ANT* in inflorescences of the *4xdella pGAI:gai-1-GR* line after 24 h of treatment with mock or 5 µM DEX. In (A) and (B), data were normalized to *UBQ10* (*At4g05320*) in L*er* or mock, respectively. Data are presented as the mean ± SD.

Development • Supplementary information

A

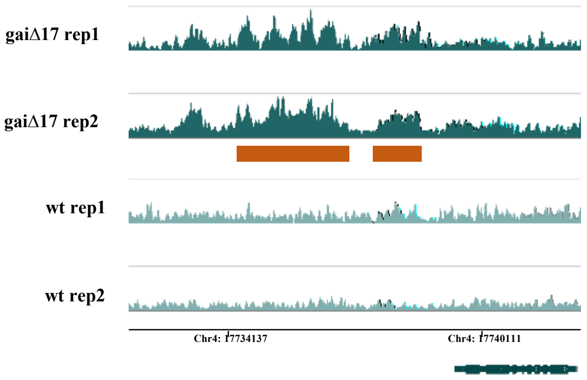

B

Gene symbol	Gene name	Chromosome	Strand	Distance to TSS
AT4G37750	ANT	chr4	+	-3961 pb
AT4G37750	ANT	chr4	+	-1256 pb

Fig. S7. GAI could bind to two domains in the promoter region of *ANT*. (A) ChIP-Seq profiles for GAI are depicted in two biological replicates for *pGAI:gaiΔ17-3xYPet* (gaiΔ17 rep1 and 2) and L*er* (wt rep1 and 2) inflorescences. Regions detected as reproducible peaks are illustrated as orange bars. (B) *ANT* gene model and chromosome position of the two putative binding sites are shown. Data were obtained from Barro-Trastoy *et al.* (2022).

Development • Supplementary information

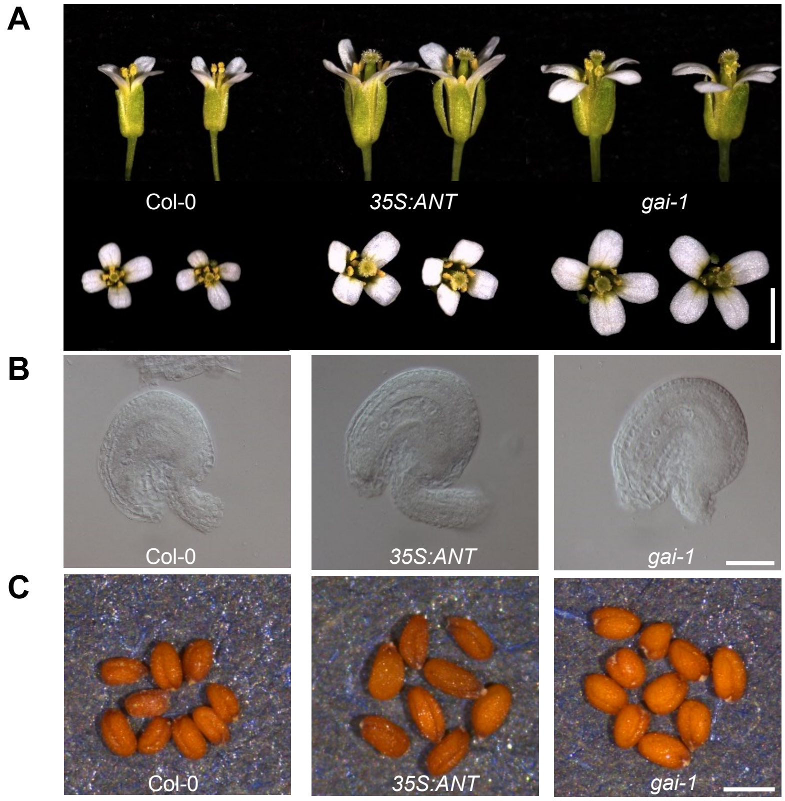

Fig. S8. Both *gai-1* and *35S:ANT* show larger floral organs.(A) Images of Col-0, *35S:ANT*, and *gai-1* flowers at anthesis stage in lateral (upper images) and zenithal (lower images) views. (B) Cleared mature ovules of Col-0, *35S:ANT*, and *gai-1*. (C) Mature seeds of Col-0, *35S:ANT*, and *gai-1*. Scale bars represent 2 mm in (A), 50 µm in (B), and 500 µm in (C).

Development • Supplementary information

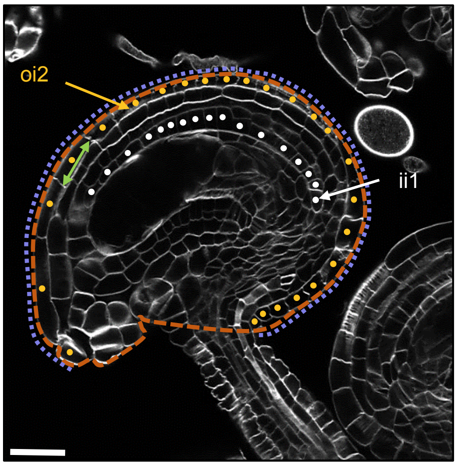

Fig. S9. Illustrative image and diagram of a mature L*er* ovule using a confocal image. The ovule was cleared and the cell walls were stained with Calcofluor White. Ovule area and oi2 layer length were estimated by measuring, in optical sections (as shown), the area enclosed by the orange dashed line and the length of the violet dotted line using ImageJ software. The yellow and white dots label each of the cells in the oi2 and ii1 layers, respectively. The average cell size of the oi2 layer cells (green arrow) was determined by dividing the oi2 layer length by the cell number. Scale bar represents 20 µm.

Development • Supplementary information

Table S1. Primers for genotyping and qPCR.

**Genotyping of *della* mutants**		
**Oligo name**	**Oligo sequence**	**Reference**
gai-1 Fw	GATCCGACATTGAAGGAAAAACC	This work
gai-1 Rv	TTGTAGTATACGTATCTCCTCCG	This work
gaiT6_wt/mut_Fw	CTAGATCCGACATTGAAGGA	Dill and Sun, 2001
gaiT6_wt_Rv	AGCATCAAGATCAGCTAAAG	Dill and Sun, 2001
gaiT6_mut_Rv	TCGGTACGGGATTTTCGCAT	Dill and Sun, 2001
rga24_wt/mut_Fw	CAAAAGTTGTTCCGGCGGTT	Lee et al., 2002
rga24_wt_Rv	GGTGATTTTCACGGTGGTTG	Lee et al., 2002
rga24_mut_Rv	GGTTTCATCCCTTTGACGCCAAACA	Lee et al., 2002
rgl1-1_wt/mut_Fw	AAGCTAGCTCGAAACCCAAAT	Lee et al., 2002
rgl1-1_wt_Rv	CCACAGAGCGCGTAGAGGATAAC	Lee et al., 2002
rgl1-1_mut_Rv	CATGGGCTGGGCCTCAGTG	Lee et al., 2002
rgl2-1_wt/mut_Fw	GCTGGTGAAACGCGTGGGAACA	Lee et al., 2002
rgl2-1_wt_Rv	ACGCCGAGGTTGTGATGAGTG	Lee et al., 2002
rgl2-1_mut_Rv	CCGGTATATCCCGTTTTGG	Lee et al., 2002
**qPCR analysis**		
qCYCB11 Fw	TTGCTGCTCGAGAGAAGAAGGCTA	Weimer et al., 2016
qCYCB11 Rv	ACATATTCCACAGCTGCGAGGTCA	Weimer et al., 2016
qCYCB12 For1	TACATTGCAGTTCCACACCGGCTA	Weimer et al., 2016
qCYCB12 Rev1	TAGCAACACCTCCATTCTCTGCCT	Weimer et al., 2016
qCYCB14 Fw	CCGAAGAATGTTGCAGGACATGGA	Weimer et al., 2016
qCYCB14 Rv	TTGTCTGCTGCTGTGGTTGTTTCG	Weimer et al., 2016
GA20ox1 Fw	CTTCCATCAACGTTCTCGAGC	This work
GA20ox1 Rv	GGTTTTGAAGGTCGATGAGAGG	This work
qANT Fw	GCGTTACAAGACATAGATGGA	This work
qANT Rv	TGCAACATATTCTTGTCTAGTC	This work
qUBQ10 Fw	GGCCTTGTATAATCCCTGATGAATAAG	Czechowski et al., 2005
qUBQ10 Rv	AAAGAGATAACAGGAACGGAAACATAGT	Czechowski et al., 2005

Development • Supplementary information

Table S2. Primers for construction of *pGAI:GAI-3xYPet*.

**Recombineering of *pGAI:GAI-3xYPeT***		
**Tagging 3xYPet at Ct of GAI[1]**		
**Oligo name**	**Oligo sequence**	**Reference**
GAI_NTest_Fw	CTAAGCAGTCCTAACCGATCCCC	Barro-Trastoy et al, 2022
GAI_NTest_Rev	CTGATTGAGAATCGCGTCACCGGG	Barro-Trastoy et al, 2022
GAI_NRec_Fw	GAAAAACCTTTTAGATCCATCTCTGAAAAAAAACCAACCATGGGAGGTGGAGGTGGAGCT	Barro-Trastoy et al, 2022
GAI_NRec_Rev	AGTCTTCTTATCTTGATGATGATGATGATGATGATCTCTCTTGGCCCCAGCGGCCGCAGC	Barro-Trastoy et al, 2022
**Trimming of genomic clones[1]**		
GAI_TrimLeft	TATGAGAATAATGAGAAAACCACTTTCCCAAATTGCTTTTTTACCAATGCTTAATCAGTG	Barro-Trastoy et al, 2022
GAI_TrimRight	TTACTCTGATTCTAACAACAAAAATCCCAAACCAAACATATAGGAACTTCCCCCTCTTGG	Barro-Trastoy et al, 2022
GAI_TrimLeft_Test	TTCCCATATGTCCACGTCAG	Barro-Trastoy et al, 2022
GAI_TrimRight_Test	TCTTTCTGAACGACCGGTTT	Barro-Trastoy et al, 2022
[1], underlined, sequence of universal adaptors.		

Development • Supplementary information
