# Supplementary material for: DELLA proteins positively regulate seed size in Arabidopsis
Source: Development. 2023 Aug 9;150(15):dev201853. doi: 10.1242/dev.201853 (PMC10445750; doi:10.1242/dev.201853)
Supplement: Supplementary file 1 [file develop-150-201853_review_history.pdf]

Development

DELLA proteins positively regulate seed size in *Arabidopsis*

Maria Dolores Gomez, Isabel Cored, Daniela Barro-Trastoy, Joaquin Sanchez-Matilla, Pablo Tornero and Miguel A. Perez-Amador

DOI: 10.1242/dev.201853

Editor: Yka Helariutta

Review timeline

Original submission:	6 April 2023
Editorial decision:	30 May 2023
First revision received:	8 June 2023
Accepted:	3 July 2023

Original submission

First decision letter

MS ID#: DEVELOP/2023/201853

MS TITLE: DELLA proteins positively regulate seed size in Arabidopsis

AUTHORS: Maria Dolores Gomez, Isabel Cored, Daniela Barro-Trastoy, Joaquin Sanchez-Matilla, Pablo Tornero, and Miguel A Perez-Amador

I have now received all the referees' reports on the above manuscript, and have reached a decision. The referees' comments are appended below, or you can access them online: please go to BenchPress and click on the 'Manuscripts with Decisions' queue in the Author Area.

As you will see, the referees express considerable interest in your work, but have some significant criticisms and recommend a substantial revision of your manuscript before we can consider publication. If you are able to revise the manuscript along the lines suggested, which may involve further experiments, I will be happy receive a revised version of the manuscript. Your revised paper will be re-reviewed by one or more of the original referees, and acceptance of your manuscript will depend on your addressing satisfactorily the reviewers' major concerns. Please also note that Development will normally permit only one round of major revision. If it would be helpful, you are welcome to contact us to discuss your revision in greater detail. Please send us a point-by-point response indicating your plans for addressing the refereeâ€™s comments, and we will look over this and provide further guidance.

Please attend to all of the reviewers' comments and ensure that you clearly highlight all changes made in the revised manuscript. Please avoid using 'Tracked changes' in Word files as these are lost in PDF conversion. I should be grateful if you would also provide a point-by-point response detailing how you have dealt with the points raised by the reviewers in the 'Response to Reviewers' box. If you do not agree with any of their criticisms or suggestions please explain clearly why this is so.

Reviewer 1

Advance summary and potential significance to field

DELLA proteins positively regulate seed size in Arabidopsis Maria Dolores Gomez, Isabel Cored, Daniela Barro-Trastoy, Joaquin Sanchez-Matilla, Pablo Tornero, and Miguel A. Perez-Amador.

© 2023. Published by The Company of Biologists under the terms of the Creative Commons Attribution License (https://creativecommons.org/licenses/by/4.0/).

In this manuscript the authors provide evidences that DELLA proteins contribute to seed size control as a DELLA gain-of-function mutant produce larger seeds with increase cell number in ovule integuments. This activity may rely on AINTEGUMENTA as GAI promotes its transcriptional activation and that over-expression of ANT produces similar seed phenotype.

Overall I think the quality of the writing and the figures are very good. This is an interesting paper demonstrating a role for DELLA proteins and more generally GA in the control of seed size which is a valuable trait for crop improvement. The molecular mechanism proposed by the author relies on the trans-activation activity of GAI and the activation of the expression of the ANT transcription factor. This paper will be of interest for a wide readership.

Comments for the author

Minor comments:
- The authors nicely show that gai-1 mutant produces larger seeds that the WT and that the gaiT6 loss-of-function mutant has smaller seeds. Other single della mutants also showed seed size phenotypes. As gid mutant and PBZ, an inhibitor of GA synthesis show an increase in seed size, the authors suggest that The greater increase in seed size following PBZ treatment could be due to the simultaneous stabilization of all DELLA proteins . I was wondering whether the authors have observed multiple combinaisons of DELLA mutants to precise the relative contribution of the different DELLA to this seed phenotype. Is seed size even smaller than gaiT6 in double or triple della mutant combinaison? or on the other hand, can we get seeds bigger than gai-1 in gai-1 rgl2Δ17 double mutant for instance?
- Figure 4: The authors claims that pCYCB1;2:Dbox-GFP expression is increased in gai-1 compare to the WT both in signal intensity and number of GFP positive cells. Can the authors provide some quantitative data on that point?
- I understand that as ANT null mutant do not produce viable ovules or seeds, it is not possible to perform genetic assay to confirm that ANT and GAI act in the same pathway but I was wondering whether gaiT6 plants seed size is impacted by ANT over-expression? If the seed size of 35S::ANT and 35S::ANT gaiT6 are comparable, this could reinforce the idea that GAI positively regulates ANT.
- In the discussion, the authors rule out the implication of BZR1 in this process based on the fact the bzr1-1D gain-of-function mutant has both an increased cell proliferation and cell expansion of the integuments cells compare to wild type. As BZR1 is controlling cell expansion through molecular complex with other regulators which is regulated by GA and DELLA, it possible that the control of cell expansion and cell division in the integument relies on two different molecular mechanisms (trans-activation versus sequestration). Even if the identification of the transcription factor interacting with GAI to allow expression of ANT is out of the scope of this paper for me, can the other discuss this possibility in the discussion of the paper.
- line 267 CYCB1;1, CYCB1;2, and CYCB1;4 enhanced their expression in siliques… and line 296 ANT enhanced its expression in gai-1 siliques… I would prefer for instance, ANT expression level is increased in gai-1 siliques as, as I understand, ANT is not activating its own expression in gai-1. Please clarify this point for line 267 and 296.
- Legend of the Figure 4 is not completely describing the data presented. The title describes only the data in the Figure4-F panel.
- Line 41: organ growth, in the ovule integuments of gai-1.Overall, our results point to DELLA… missing space before Overall .
- line 430: In the Materials and Methods section, name of single mutant should be in italics (line 434-435 and 440).

Reviewer 2

Advance summary and potential significance to field

Demonstration that DELLA proteins acting maternally influence seed size in Arabidopsis contributes to an understanding of the regulation of cell proliferation in the integuments and reinforces the importance of AINTEGUMENTA in the maternal control of seed deveolpment.

© 2023. Published by The Company of Biologists under the terms of the Creative Commons Attribution License (https://creativecommons.org/licenses/by/4.0/).

Comments for the author

The manuscript of Gomez et al., examines the influence of DELLA proteins on seed size in Arabidopsis. The use of GA signalling gain- and loss of function mutants revealed a correlation between DELLA activity and seed size without modifying seed number or reserve composition. The authors show DELLA acts maternally to increase ovule size by increasing cell division in outer and inner integument layers and detail DELLA expression in developing ovule domains using reporter transgene imaging. They document increased expression of B-cyclins in gai-1 in support of the correlation between integument cell division and DELLA expression leading to increased integument cell proliferation. The authors implicate ANTEGUMENTA in mediating DELLA function noting the similarity of integument phenotypes between ANT overexpression and the gai-1 gain of function mutant as well as the enhanced transcription of ANT in gai-1 or by production of ANT after gai-1 induction.

The experimentation is convincing and the interpretation is appropriate. The evidence presented is novel, supporting DELLA proteins as determinants of Arabidopsis seed size by increasing integument cell number during ovule development. The involvement of ANT in DELLA regulation of seed size is plausible but additional experimentation to demonstrate regulation of ANT by GAI is necessary, perhaps by examining phenotypes of knock down alleles such as ant-T, screening for ANT promoter binding factors and rigorous GAI 2-hybrid screens with transcription factor libraries.

Comments:
Figure 3G-H: I have impression that reporter expression stronger in II1 and OI1 whereas cell counts were higher for OI2 Fig2C. Please clarify.
Figure 4G-H: Comparison between Ler and gai-1 fluorescence imaging is somewhat subjective. How many observations?
Figure 6: Model is speculative, the essence of the study is the relation between DELLA and ANT whereas evidence for DELLA interactors/targets and possible co-regulation by GAI promoter binding by ANT not examined. Perhaps present a more general model including MEE45 and ARF2 regulation?

Minor issues:
156: substitute “…was associated with…” for “caused”
221: fig S4A-C Integument cell size, length increases number increases but size decreases?
289: elaborate ‘indirect’ GAI binding sites 345: insert “…potentially powerful...”
365: substitute “…associated with an…” for “…likely through…”
371-372 and 398: are these observations mutually compatible in the present study?
393: substitute “… the ANT-YPet protein level is greater in…” for ‘protein increases its levels’
818: suppress “Ratio”

First revision

<u>Author response to reviewers' comments</u>

RESPONSE TO REVIEWERS:

In the revised manuscript, new text is indicated in blue, whereas eliminated text is marked by double strikethrough. Following, we provide our point-to-point response to reviewers:

Reviewer 1 Comments for the Author:
Minor comments:
- The authors nicely show that gai-1 mutant produces larger seeds that the WT and that the gaiT6 loss-of- function mutant has smaller seeds. Other single della mutants also showed seed size phenotypes. As gid mutant and PBZ, an inhibitor of GA synthesis show an increase in seed size, the authors suggest that The greater increase in seed size following PBZ treatment could be due to the simultaneous stabilization of all DELLA proteins . I was wondering whether the authors have observed multiple combinaisons of DELLA mutants to precise the relative contribution of the

© 2023. Published by The Company of Biologists under the terms of the Creative Commons Attribution License (https://creativecommons.org/licenses/by/4.0/).

different DELLA to this seed phenotype. Is seed size even smaller than gaiT6 in double or triple della mutant combinaison? or on the other hand, can we get seeds bigger than gai -1 in gai-1 rgl2Δ17 double mutant for instance?
Response: As nicely pointed by the reviewer, double and triple *della* mutants also show reduced seed size. We did not include this data in the original version as the single mutants already show a clear phenotype. Following the suggestion of the reviewer, we have included a new panel in Figure S2 (new S2A), where we show the reduced seed size of *3xdella* and the *4xdella* mutants. Regarding the gain-of- function mutants, we generated several double mutants, but in this case, the strong defects in plant development due to stable DELLA proteins hamper the determination of the seed phenotype. For example, the *gai-1 rga□17* mutant (*rga□17* from *pRGA:GFP-rga□17* line) plant has extremely late flowering, and defects in flower and pistil development and fertility, *gai-1 rgl2□17* (*rgl2□17* from Gomez et al., 2019) is impaired in germination, whereas *gai-1 rgl1□17* (*rgl1*Δ17 Gomez et al., 2020) has strong defect in plant development and flowering. Besides, we also observed increased seed size in PBZ-treated L*er* plants, where protein levels of all DELLAs should be increased (Figure S2C,E). These are L*er* plants treated upon bolting with 1 μM PZB, a non-saturated dose, so we avoid defects in germination, plant establishment, or flowering time observed in the double dominant mutants.

- Figure 4: The authors claims that pCYCB1;2:Dbox-GFP expression is increased in gai-1 compare to the WT both in signal intensity and number of GFP positive cells. Can the authors provide some quantitative data on that point?
Response: All our confocal analyses are done using at least 3 batches of plants, grown at different times. From each, at least 5 inflorescences (one inflorescence by plant) are analyzed to look for specific developmental stages of ovule development. Overall at least 12-15 good quality images are obtained (a stack of images to detect all GFP-positive nucleus in each ovule, not in a single confocal plane). We have determined the number of GFP-positive nuclei from these images to quantify the increase in cyclin expression: gai-1 has a 70% increase in GFP-positive nuclei when compared to L*er* (approximately 3 nuclei in L*er* and 5 in *gai-1*). The new data is included in the text and in the figure legends.

- I understand that as ANT null mutant do not produce viable ovules or seeds, it is not possible to perform genetic assay to confirm that ANT and GAI act in the same pathway but I was wondering whether gaiT6 plants seed size is impacted by ANT over-expression? If the seed size of 35S::ANT and 35S::ANT gaiT6 are comparable, this could reinforce the idea that GAI positively regulates ANT.
Response: Testing of *35S:ANT* and *35S:ANT gaiT6* would be important. Unfortunately, we do not have this double mutant at this moment. The main reason is that *35S:ANT* is in Col0, whereas *gaiT6* is in L*er* backgrounds. Therefore, generation of this mutant requires more than one year (initial cross and at least 3x backcrosses). We are generating this mutant to show the data in the context of the following paper where we plan to identify and describe the potential GAI interacting protein that is responsible of the direct regulation of *ANT*, as well as the identification of other GAI-targets during ovule development that would be involved in the increased ovule and seed size. We are currently embarked in the identification of GAI interacting proteins specifically in the ovule integuments by a TurboID approach (see full response to reviewer#2 below).

- In the discussion, the authors rule out the implication of BZR1 in this process based on the fact the bzr1 -1D gain-of-function mutant has both an increased cell proliferation and cell expansion of the integuments cells compare o wild type. As BZR1 is controlling cell expansion through molecular complex with other regulators which is regulated by GA and DELLA, it possible that the control of cell expansion and cell division in the integument relies on two different molecular mechanisms (trans-activation versus sequestration). Even if the identification of the transcription factor interacting with GAI to allow expression of ANT is out of the scope of this paper for me, can the other discuss this possibility in the discussion of the paper.
Response: We agree that this idea is worth to be included in the discussion. We have proceeded accordingly and modified the discussion.

- line 267 CYCB1;1, CYCB1;2, and CYCB1;4 enhanced their expression in siliques… and line 296 ANT enhanced its expression in gai-1 siliques… I would prefer for instance, ANT expression level is increased in gai-1 siliques as, as I understand, ANT is not activating its own expression in gai-1. Please clarify this point for line 267 and 296.

© 2023. Published by The Company of Biologists under the terms of the Creative Commons Attribution License (https://creativecommons.org/licenses/by/4.0/).

Response: Nice suggestion. Changes in the text are done as indicated to reduce ambiguity.

- Legend of the Figure 4 is not completely describing the data presented. The title describes only the data in the Figure4-F panel.

Response: Figure title has been changed to reflect the data presented. The new tittle is '*ANT* and *CYC* gene expression are increased in *gai-1* ovules'.

- Line 41: organ growth, in the ovule integuments of gai-1.Overall, our results point to DELLA… missing space before Overall .

Response: Change done as indicated.

- line 430: In the Materials and Methods section, name of single mutant should be in italics (line 434-435 and 440).

Response: Thanks for the tip, the mutants are always in italics through the text. We miss it here probably during handling of the text. Changes in the text are done as indicated.

Reviewer 2 Comments for the Author:

The experimentation is convincing and the interpretation is appropriate. The evidence presented is novel, supporting DELLA proteins as determinants of Arabidopsis seed size by increasing integument cell number during ovule development. The involvement of ANT in DELLA regulation of seed size is plausible but additional experimentation to demonstrate regulation of ANT by GAI is necessary, perhaps by examining phenotypes of knock down alleles such as ant-T, screening for ANT promoter binding factors and rigorous GAI 2-hybrid screens with transcription factor libraries.
Response: We agree that it is crucial to show the direct relationship between GAI and ANT and look for the GA-interacting protein involved. Unfortunately, in our experimental conditions, we have not observed any ovule/seed phenotype in *ant-T* mutant plants. Therefore, we have not used this line to address the direct regulation of GAI-ANT. We have a list of transcriptional regulators that are putative RGA and GAI-interacting proteins, in a Y2H assay (https://doi.org/10.1007/978-1-4939-7871-7_9) using a platform with over 1200 transcriptional regulators of Arabidopsis. This list of genes lacks the specificity as we are looking for interactors in a specific tissue and developmental stage. Similar criticism can be done to the search for ANT promoter binding factors. Both GAI and ANT are being expressed in different tissues during plant development. To cope with this, we are currently carrying out a TurboID approach to identify GAI-interacting proteins in the integuments of developing ovules. For this we have generated several lines that express the fusion of GAI-TurboID (TurboID can add biotin to proteins situated in close proximity) under the control of the *INO* (*INNER-NO-OUTER*) promoter that directs the expression specifically to the integuments during ovule development, as well as all the control lines needed to reduce false-positives. Upon biotin treatment in inflorescences, only proteins that are very close to GAI in integuments will be biotinylated by TurboID. Later, these proteins can be isolated and identified by MS. We hope that this strategy will provide experimental evidence of the genetic components integrating the molecular network involved in the control of the GAI-mediated ovule and seed.

Comments:

Figure 3G-H: I have impression that reporter expression stronger in II1 and OI1 whereas cell counts were higher for OI2 Fig2C. Please clarify.
Response: Indeed, it seems that GFP signal is stronger in II1 and oi1, and lower in ii2 or oi2 in Figure 3G- H. It must be noted that cell division is reduced at the developmental stage shown in 3G. Moreover, *gai-1* expression is stronger in the oi2 at earlier developmental stages, such as that shown in Figure 3F, when cell division is very active. Finally, it is not known whether cells of the inner integument could be able to activate division in the outer integument, which would be required

© 2023. Published by The Company of Biologists under the terms of the Creative Commons Attribution License (https://creativecommons.org/licenses/by/4.0/).

for coordinated growth of all cell layers.

Figure 4G-H: Comparison between Ler and gai-1 fluorescence imaging is somewhat subjective. How many observations?

Response: As indicated above, we have compared these genotypes from different (at least three) batches of plants, growth at different moments. From each set of plants, we usually obtain good quality confocal images from at least 5 inflorescences. The selected image is always representative of the whole set of images. In the case of the *CYC* expression, we have used these good-quality images to count the number of GFP-positive nucleus as suggested by reviewer#1. We indicate this in Methods and in the figure legend.

Figure 6: Model is speculative, the essence of the study is the relation between DELLA and ANT whereas evidence for DELLA interactors/targets and possible co-regulation by GAI promoter binding by ANT not examined. Perhaps present a more general model including MEE45 and ARF2 regulation?

Minor issues:

Response: We agree that this figure is speculative as it shows the current working model of the possible molecular mechanism integrating the DELLA and ANT in the control of ovule and seed size. Following the suggestion, we have modified Figure 6 to include the direct regulation of the expression of *ANT* by MEE45 and ARF2.

156: substitute "…was associated with…" for "caused"

Response: Changes in the text are done as indicated.

221: fig S4A-C Integument cell size, length increases number increases but size decreases?

Response: We understand that the current version of the figure S4 may be misleading. The panel A indicates the length of the whole oi2 cell layer, whereas panels B and C represent the average cell number and size, respectively, in the same oi2 cell layer. The whole cell layer increases, due mainly to strong increase in cell number, although these cells are slightly smaller. To avoid confusion, the figure legend and the Y-axe labels have been modified.

289: elaborate 'indirect' GAI binding sites

Response: We have modified the text to clarify this issue. In the introduction we have already stated that DELLA proteins do not contain a DNA binding domain and hence they regulate transcription by direct binding to transcription factors (Vera-Sirera et al., 2015; Daviere and Achard, 2016).

345: insert "…potentially powerful..."

365: substitute "…associated with an…" for "…likely through…"

Response: Changes in the text are done as indicated.

371-372 and 398: are these observations mutually compatible in the present study?

Response: We agree that both statements seem contradictory, but they are not. A delay in embryonic development has been observed in some (but not all) mutants that have larger seeds. We therefore wanted to check whether this was also the case in *gai-1* and found that *gai-1* embryonic development is not delayed compared to WT. This would be compatible with the fact that *gai-1* seeds are larger due to increased expression of ANT, as ANT prolongs cell proliferation at very early stages of ovule development, before fertilization occurs. We would like to emphasize that the mature *gai-1* ovule is already larger than WT ovule, which may explain that the embryo development is not delayed.

© 2023. Published by The Company of Biologists under the terms of the Creative Commons Attribution License (https://creativecommons.org/licenses/by/4.0/).

393: substitute "… the ANT-YPet protein level is greater in…" for 'protein increases its levels'

818: suppress "Ratio"

Response: Changes in the text are done as indicated.

Sincerely,
María D. Gomez

Second decision letter

MS ID#: DEVELOP/2023/201853

MS TITLE: DELLA proteins positively regulate seed size in Arabidopsis

AUTHORS: Maria Dolores Gomez, Isabel Cored, Daniela Barro-Trastoy, Joaquin Sanchez-Matilla, Pablo Tornero, and Miguel A Perez-Amador
ARTICLE TYPE: Research Article

I am happy to tell you that your manuscript has been accepted for publication in Development, pending our standard ethics checks.

Reviewer 1

Advance summary and potential significance to field

In this manuscript the authors provide evidences that DELLA proteins contribute to seed size control as a DELLA gain-of-function mutant produce larger seeds with increase cell number in ovule integuments. This activity may rely on AINTEGUMENTA as GAI promotes its transcriptional activation and that over-expression of ANT produces similar seed phenotype.

Comments for the author

This last version of the manuscript together with the added data takes into account all of my comments therefore I believe it's ready for publication.

Reviewer 2

Advance summary and potential significance to field

A demonstration of correlation between DELLA activity and seed size mediated through an increase in ovule size resulting from increased cell proliferation in outer and inner integument layers. Development of conceptual model implicating AINTEGUMENTA as a nexus for control of organ growth.

Comments for the author

The revised manuscript of Gomez et al., has addressed the minor issues raised in my initial review. There are now a few points to correct and elaborate in version 2.
Lines 267-269: The statement 'The number of GFP positive…respectively)' refers to which panels in Figure 4?

© 2023. Published by The Company of Biologists under the terms of the Creative Commons Attribution License (https://creativecommons.org/licenses/by/4.0/).

Line 290: replace ‘throught’ by through Lines 311 – 313: Are there other alleles of ANT that could provide insight into the regulation by DELLA?

Line 425: suppress sentence ‘Finally…ARF24’ this is unnecessary since this is already elaborated in the preceding text Lines 410-411

The model for DELLA control over ovule and seed size mediated through ANT remains speculative.

© 2023. Published by The Company of Biologists under the terms of the Creative Commons Attribution License (https://creativecommons.org/licenses/by/4.0/).
